# Chemical Decorations of “MARs” Residents in Orchestrating Eukaryotic Gene Regulation

**DOI:** 10.3389/fcell.2020.602994

**Published:** 2020-12-21

**Authors:** Tanaya Roychowdhury, Samit Chattopadhyay

**Affiliations:** ^1^Department of Biological Sciences, Birla Institute of Technology & Science, Pilani, India; ^2^Cancer Biology and Inflammatory Disorder Division, Indian Institute of Chemical Biology, Kolkata, India

**Keywords:** post-translation modification (PTM), S/MAR-binding protein, gene regulation, disease, chromatin 3D architecture

## Abstract

Genome organization plays a crucial role in gene regulation, orchestrating multiple cellular functions. A meshwork of proteins constituting a three-dimensional (3D) matrix helps in maintaining the genomic architecture. Sequences of DNA that are involved in tethering the chromatin to the matrix are called scaffold/matrix attachment regions (S/MARs), and the proteins that bind to these sequences and mediate tethering are termed S/MAR-binding proteins (S/MARBPs). The regulation of S/MARBPs is important for cellular functions and is altered under different conditions. Limited information is available presently to understand the structure–function relationship conclusively. Although all S/MARBPs bind to DNA, their context- and tissue-specific regulatory roles cannot be justified solely based on the available information on their structures. Conformational changes in a protein lead to changes in protein–protein interactions (PPIs) that essentially would regulate functional outcomes. A well-studied form of protein regulation is post-translational modification (PTM). It involves disulfide bond formation, cleavage of precursor proteins, and addition or removal of low-molecular-weight groups, leading to modifications like phosphorylation, methylation, SUMOylation, acetylation, PARylation, and ubiquitination. These chemical modifications lead to varied functional outcomes by mechanisms like modifying DNA–protein interactions and PPIs, altering protein function, stability, and crosstalk with other PTMs regulating subcellular localizations. S/MARBPs are reported to be regulated by PTMs, thereby contributing to gene regulation. In this review, we discuss the current understanding, scope, disease implications, and future perspectives of the diverse PTMs regulating functions of S/MARBPs.

## Introduction

**Figure 1:**
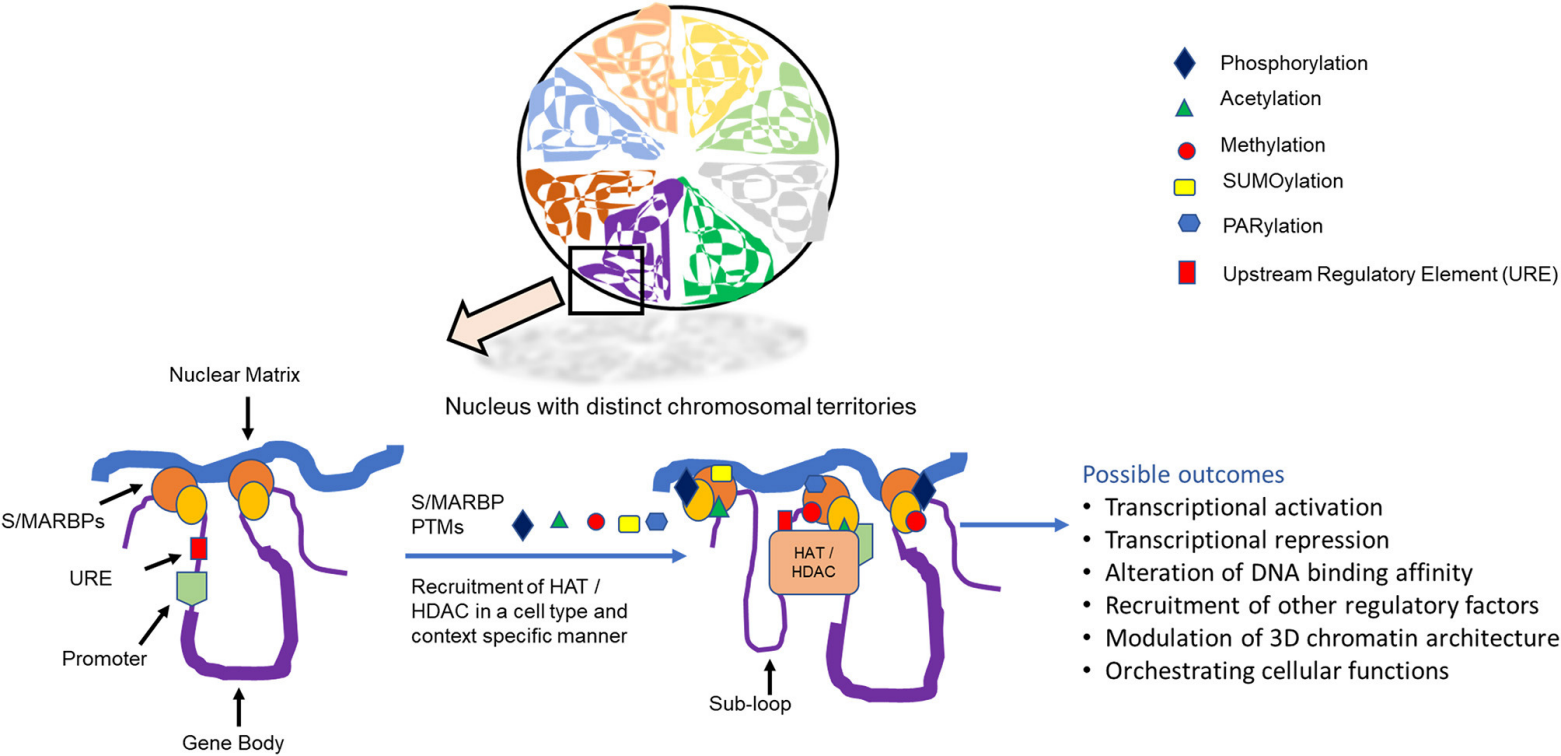
Distinct chromosomal territories showing scaffold/matrix attachment region binding proteins (S/MARBPs) that mediate chromatin attachment and with matrix can be regulated by post-translational modifications, leading to various outcomes.

The nucleus in eukaryotes is known to harbor a three-dimensional (3D) protein network referred to as the nuclear matrix or scaffold. This scaffold provides the nucleus with a framework that aids in the maintenance of overall size and shape. It was defined as the biochemical fraction containing mainly DNA, RNA, and different non-histone proteins that remained after treatment with detergent, salt, and nucleases (Berezney and Coffey, 1974). Experimental findings support the notion that eukaryotic chromatin is organized in the form of independent loops (Ea et al., 2015) This loop structure is essential for chromosomal packaging, replication, transcription, and splicing (Boulikas, 1996; Girard-Reydet et al., 2004; Kulkarni et al., 2004; Coffman et al., 2006; Girod et al., 2007; Wang et al., 2007) and is visible as a halo of DNA anchored to the densely stained nuclear scaffold after histone extraction (Gerdes et al., 1994; Fukuda, 1999; Goetze et al., 2003). These loops are anchored to the matrix via specific DNA sequences known as matrix attachment region or scaffold-associated region, collectively termed scaffold/matrix attachment regions (S/MARs) (Figure 1). These regions are AT-rich and contain other AT-rich sequence motifs. They are about 200 bp long and frequently found close to the *cis*-acting regulatory sequences. Several characteristics have been proposed for S/MAR sequences like their enrichment in DNase I-hypersensitive sites, inverted repeats, polypurine stretches, DNA unwinding elements, binding sites for replication initiator proteins, homo-oligonucleotide repeats (like TTT, CCC, and AAA), motifs having potential to form triple helix, and left-handed structures. S/MARs are functionally conserved, and experiments have validated the binding of animal S/MARs to plant nuclear scaffolds and vice versa (Fukuda, 1999; Wang et al., 2004).

The mechanisms by which the different S/MAR functions are still under investigation, as it would be also dependent on the *trans*-acting proteins that interact with it. Several proteins have been isolated in eukaryotes, which bind to these S/MARs and hence are termed S/MAR-binding proteins (S/MARBPs). These proteins can interact with different machineries of replication, transcription, repair, epigenetic modulators, splicing, etc., and thereby orchestrate the various nuclear events. An exact relationship between the structure and function of S/MARBPs is yet not available. Very few of the MARBPs like CTCF, SATB1 and poly (ADP-ribose) polymerase (PARP) 1 have their structures solved partially. Most of the time, the structure of the DNA-binding domain either alone or in association with some DNA sequence or inhibitor has been solved using either X-ray crystallography or in-solution NMR. However, the obtained information does not elucidate the structure–function relationship conclusively; their functions can be modulated through different post-translational modifications (PTMs) (Figure 1). PTM can be defined as covalent addition of functional group to a protein after translation in response to a cellular stimulus. This is a universal process that regulates cellular functions of proteins in general.

## Scaffold/Matrix Attachment Region-Binding Proteins and Their Functions

Majority of S/MARBPs have been found as constituents of nuclear matrices. From the rat liver extract, 12 proteins were identified, and six of them (viz., matrins D, E, F, and 4, and lamins A and C) were identified as DNA-binding proteins (Hakes and Berezney, 1991; Nakayasu and Berezney, 1991). The structurally related proteins desmins and NuMA also were found to bind specifically to S/MARs *in vitro*. These interactions mainly involved two structural features. One involved the single stranded regions, and the other was the minor groove of the double-stranded S/MAR (Luderus et al., 1994). Topoisomerase II preferentially binds S/MAR DNA at about one dimer per 200 bp of DNA (Adachi et al., 1989). HMGY(I) proteins have been reported to bind, bend, and unwind DNA supercoils. They also interact with other protein partners, which in turn help in the regulation of transcription (Reeves and Beckerbauer, 2001). B-cell regulation of immunoglobulin heavy chain or Bright is B-cell-specific protein identified for its ability to upregulate transcription of immunoglobulin gene by 3- to 7-fold in activated B cells. It binds to AT-rich regions (Webb, 2001). NMP1 and NMP2 are two S/MARBPs that bind to osteocalcin gene promoter in osteoblasts. NMP1 is more ubiquitous in nature and is found in both matrix and non-matrix nuclear compartments, while NMP2 is more cell type specific in nature (Bidwell et al., 1993). HnRNP-U/scaffold attachment factor (SAF)-A in vertebrates binds to S/MARs as validated by *in vivo* UV crosslinking experiments. *In vitro*, it binds to both single- and double-stranded DNA, forming higher-order nucleic acid protein structures (Fackelmayer et al., 1994). SAF-B was identified as a novel S/MARBP in HeLa cells. It is expressed in all human tissues. This protein is of 96.69-kDa size and does not share much sequence homology with any other protein (Renz and Fackelmayer, 1996). SATB1 is an S/MARBP that is expressed in the thymus and binds along the minor grove with little contact with the bases (Nakagomi et al., 1994). SATB2 is closely related to SATB1 but differs by small ubiquitin-related modifier (SUMO) at two lysine residues. It is more cell type specific and binds to S/MARs of immunoglobulin micro locus in pre-B cells, where it enhances gene expression (Dobreva et al., 2003). SMAR1 was identified as a novel S/MARBP that interacted with the S/MAR beta, a region that is 400 bp upstream of Eβ enhancer of T-cell receptor β gene (Chattopadhyay et al., 2000). Cux/CDP and SATB1 were also found to be capable of binding to an S/MAR element upstream of T-cell receptor β gene enhancer. These fine-tunes the enhancer-driven receptor gene expression. However, endogenous gene expression was not altered (Chattopadhyay et al., 1998). p114 was identified in SK-BR-3 breast cancer cell line. It has a high affinity to A+T-rich S/MAR-like probe. Interestingly, it is found in human breast cancer tissues but not in normal benign tissues, benign diseases of the breast, or immortalized normal breast cell line like MCF10A (Yanagisawa et al., 1996). Bright (B-cell regulator of IgH transcription) interacts with Sp100, which is a component of promyelocytic leukemia (PML) nuclear bodies and the lymphoid restricted homolog of Sp100, LYSp100/Sp140. Both inhibit the S/MAR binding and transactivation activity of Bright. However, LYSp100/Sp140 interacts weakly and is therefore required at a considerably higher level than Sp100 for inhibiting S/MAR-binding activity (Zong et al., 2000). Nucleolin was purified from K562 erythroleukemia cells. It has high binding affinity toward various double-stranded S/MARs from different species. Nucleolin in found in both matrix and non-matrix nuclear regions (Dickinson and Kohwi-Shigematsu, 1995). High-Mobility Group (HMG) proteins also bind S/MARs as suggested by the interaction of anti-HMG antibodies with S/MARs (Ivanchenko and Avramova, 1992). Base-unpairing regions (BURs) are specialized S/MARs. These are smaller regions found in S/MARs, which have high affinity for isolated nuclear framework *in vitro*. BUR affinity chromatography was used to isolate such BUR-binding proteins from SK-BR-3 breast cancer cell line. Two such proteins were identified, DNA-dependent protein kinase (DNA-PK) and PARP (Galande and Kohwi-Shigematsu, 1999). Years of extensive research has established that wild-type p53 (WT p53) is a tumor suppressor and fine-tunes several effector pathways, thereby maintaining cellular homeostasis and genomic stability. Most of the tumors harbor p53 mutations, some abrogate normal functions, and others impart gain-of-function oncogenic properties. Mutant p53 (MUT p53) can repress or activate MUT p53-specific genes. Interestingly, MUT p53 can bind to different nonlinear DNA sequences like S/MARs. It plays a nodal role in oncogenic signaling by modulating chromatin architecture and maintaining cancer-specific transcriptome. It seems that MUT p53 has a binding affinity to non-canonical DNA structures with dependency on sequence composition (Kim and Deppert, 2007). S/MAR sequences bound by MUT p53 are rich in AT and exhibit variations of AATATATTT unwinding motif. This allows for structural flexibility and enhances chromatin dynamics and base unpairing. Introducing mutations in the unwinding motif adversely affects MUT p53 binding (Will et al., 1998). CCCTC-binding factor (CTCF) is a vertebrate 11-zinc finger (ZF) protein and was first identified as a transcriptional repressor of cMyc. Later studies confirmed that it binds genome wide in a sequence-specific manner and performs a plethora of functions including X-chromosome inactivation, transcriptional activation or repression, imprinting, and enhancer blocking (Zlatanova and Caiafa, 2009). CTCF is also an S/MARBP that helps in defining boundaries between topologically distinct functional domains of genome (Dunn et al., 2003).

S/MARBPs have been found in other eukaryotic organisms also. This suggests a conserved role for such class of proteins in maintaining genome organization. ARBP, a chicken S/MARBP, selectively binds to an S/MAR element of lysozyme gene. It is found as a component of internal nuclear network, and *in vitro* experiments suggest its role in the maintenance of chromatin architecture (Hatton and Gray, 1999). MFP1 was identified in tomato. It contains a probable transmembrane N-terminal domain and a filamentous long alpha helical domain. Comparison of primary and secondary structures with tobacco and *Arabidopsis* points to a high degree of conservation. This N-terminal domain is required for its targeting to nuclear speckles (Fujiwara et al., 2002). MAF1 is a novel S/MARBP identified also in tomato using yeast two-hybrid assay and *in vitro* experiments (Gindullis et al., 1999). Like MFP1, MAF1 is found near the nuclear periphery and matrix, thereby suggesting an interaction between these two proteins *in vivo*. A novel S/MARBP called AT-hook motif nuclear localized protein 1 (AHL1) was identified in *Arabidopsis* during a visual screening of transformants using GFP:cDNA fusions. This protein is found mainly in nucleoplasm and is concentrated at the boundary between heterochromatin and euchromatin. During M phase, it was found to be localized at the chromosomal surface. The AT-hook region was required for S/MAR-binding activity (Morisawa et al., 2000). S/MARBP1 and S/MARBP2 were identified from pea leaf cDNA library. It interacted with AT-rich S/MAR sequences (Hatton and Gray, 1999). A similar S/MARBP was identified from *Nicotiana tabacum* cells, having a molecular weight of 61.050 kDa. It was hence named NtS/MARBP61, and its C-terminal domain interacted with 12 different S/MARs (Fujiwara et al., 2002).

With continuous scientific ventures, more and more S/MARBPs have been identified, and functional screens have highlighted their importance in gene regulation and chromatin architecture maintenance. These proteins are often found with other chromatin remodelers, which ultimately regulate gene expression. Bright, for example, can transactivate gene expression from IgH enhancer Eμ. It is present in differentiated cells, where it can compete with other repressors for S/MAR site (Chattopadhyay and Pavithra, 2007). SATB1 is mainly expressed in the thymus, but it is also found in the brain and other organs. It acts by binding S/MAR sites in several genes, leading to their repression. Some examples include c-myc, interleukin-2 receptor alpha, and genes encoded by mouse mammary tumor virus (MMTV), which is a glucocorticoid-responsive retrovirus. SATB1 binds to S/MAR sites in the provirus and represses transcription from it (Liu et al., 1997; Yasui et al., 2002; Seo et al., 2005). It also regulates the class I Major Histocompatibility Complex (MHC) locus by organizing it into discrete loops and by tethering it to S/MARs (Galande et al., 2007). SAFB1 can repress estrogen receptor alpha-mediated transcription partly via interaction with other corepressors (Jiang et al., 2006). SMAR1 is expressed in all tissues but is predominant in the thymus, where it regulates the transition of T cells from double-negative (DN) to double-positive (DP) stage (Kaul-Ghanekar et al., 2005). Interestingly, SMAR1 acts as a tumor suppressor by playing a role in activating p53 via direct interaction and phosphorylation (Kaul et al., 2003). It also inhibits MDM2-mediated degradation of p53 (Pavithra et al., 2009). SMAR1 plays a role in development of lymphoid organs (Kaul-Ghanekar et al., 2005). As S/MARBPs are important regulators of chromatin architecture, which fine-tunes transcription, it is expected that these proteins would play major roles in organogenesis and development. For example, studies have shown Cux knockout mice have been found to have fewer B and T cells, delayed development of lung epithelia, growth retardation, developmental defects of hair follicles, and infertile male progeny (Tufarelli et al., 1998; Sinclair et al., 2001). During the initial phase of apoptosis, SATB1 has been seen to have altered three-dimensional distribution, which finally leads to cleavage of this protein resulting in collapse of nuclear architecture. Caspase 3 mediates cleavage of this protein, and this process seems specific to apoptosis. These observations suggest that S/MARBP proteolysis could be a general mechanism in an apoptotic cell that would expose DNA sites to endonucleases (Sun et al., 2006). Thus, it is evident that S/MARBPs play various functions to maintain nuclear architecture (Chattopadhyay and Pavithra, 2007), which in turn orchestrates several aspects of cellular homeostasis. Hence, it is important to delve deeper into the mechanisms that regulate these proteins.

## An Overview of Scaffold/Matrix Attachment Region-Binding Protein Structure–Function Relationship

Nuclear organization and architecture are important for the functional outcomes like DNA replication, transcription, and repair (Razin et al., 1996; Stein et al., 2003). It is the intricate interaction between the nuclear matrix and the chromatin that fine-tunes the different regulatory processes. S/MAR and S/MARBP interactions help in the formation of chromatin loops that bring widely separated regulatory elements together, thereby modulating gene expression (Capco et al., 1982). S/MARs in general lack sequence conservation; however, they share structural similarity (Breyne et al., 1992; Laemmli et al., 1992). With advancement of high-throughput sequencing, several genome-wide studies like ChIP-Seq, ChIA-PET, and HiC have been performed to identify the genome-wide occupancies of various S/MARBPs (Han et al., 2018). HiC studies done at a map resolution of 1 kb gave detailed information regarding the CTCF contact domains. The loci within the same contact domain displayed high levels of correlation between the histone modifications (viz., H3K4me1, H3K4me2, H3K4me3, H3K9me3, H3K27me3, H3K36me3, and H3K4me1) compared with loci present in different domains based on GM12878 cell line. Quite often, the changes in the long-range contact patterns change with changes in the chromatin states of loci in a contact domain. Similar HiC studies in seven more human cell lines (viz., HMBC, KBM7, HUVEC, IMR90, NHEK, HeLa, and K562) revealed that almost 50–75% of the peak was conserved. Therefore, the basic genome organization remains constant, but the fine-tuning takes place in a context-specific manner, which is associated with changes in the interacting partners. In most of the cases, the loops are tethered at a pair of convergent binding sites for CTCF and SMC3/RAD21. Genome organization by CTCF in large scale is mediated by specific orientation of CTCF binding sites. However, many of the short-range interactions do not follow the same rule, and often, the convergent sites are not involved in the weakly formed interactions (Rao et al., 2014). CTCF contains multiple ZF motifs and has a varying degree of affinity for DNA depending on the sequence in the canonical or less characterized non-canonical motifs. Although CTCF binds to the convergent sites, the strength of the interaction varies, and it is not yet understood why. Studies have long shown that CTCF does not work alone and has multiple protein partners (Ghirlando and Felsenfeld, 2016). The protein–protein interactions (PPIs) govern the regulatory functions of CTCF. SATB1 is another example of a well-studied S/MARBP, which has mainly been studied as a T-cell-specific genome organizer. The human and mouse SATB1 proteins share 98.3% homology. SATB1 has an N-terminal domain that harbors the nuclear localization signal followed by the PDZ domain. The N-terminal domain itself can interact with DNA motif; however, dimerization is needed for DNA-binding activity. For the higher-order loop structure formation, tetramerization is essential. The DNA-binding activity of the N-terminal domain is likely due to the CUT-like domain in it (Zelenka and Spilianakis, 2020). The CUT domain determines the affinity to DNA, and the homeodomain imparts specificity of interaction (Yamasaki et al., 2007). Although studies have revealed that SATB1 has prominence of TAATA sequence in the motif, but interestingly, SATB1 does not bind to all the TAATA motifs available. This implies that adjacent or neighboring sequence, interacting protein partners, conformation of SATB1, etc., would be important in determining the binding sites. A very recent study has identified that DNA and SATB1 interactions are mechanosensitive in nature. With the use of deep sequencing and single-cell live cell imaging, it was revealed that SATB1 preferentially binds nucleosome-rich regions and directly binds the consensus motifs within the nucleosomes. It is found that an increase in the negative torsional stress within DNA promotes SATB1 binding, and it stabilizes the BURs against melting via molecular machines (Ghosh et al., 2019). There are other S/MARBP proteins that would be partially similar in terms of function, but a rigorous discussion about those is beyond the scope of this review.

**Figure 2:**
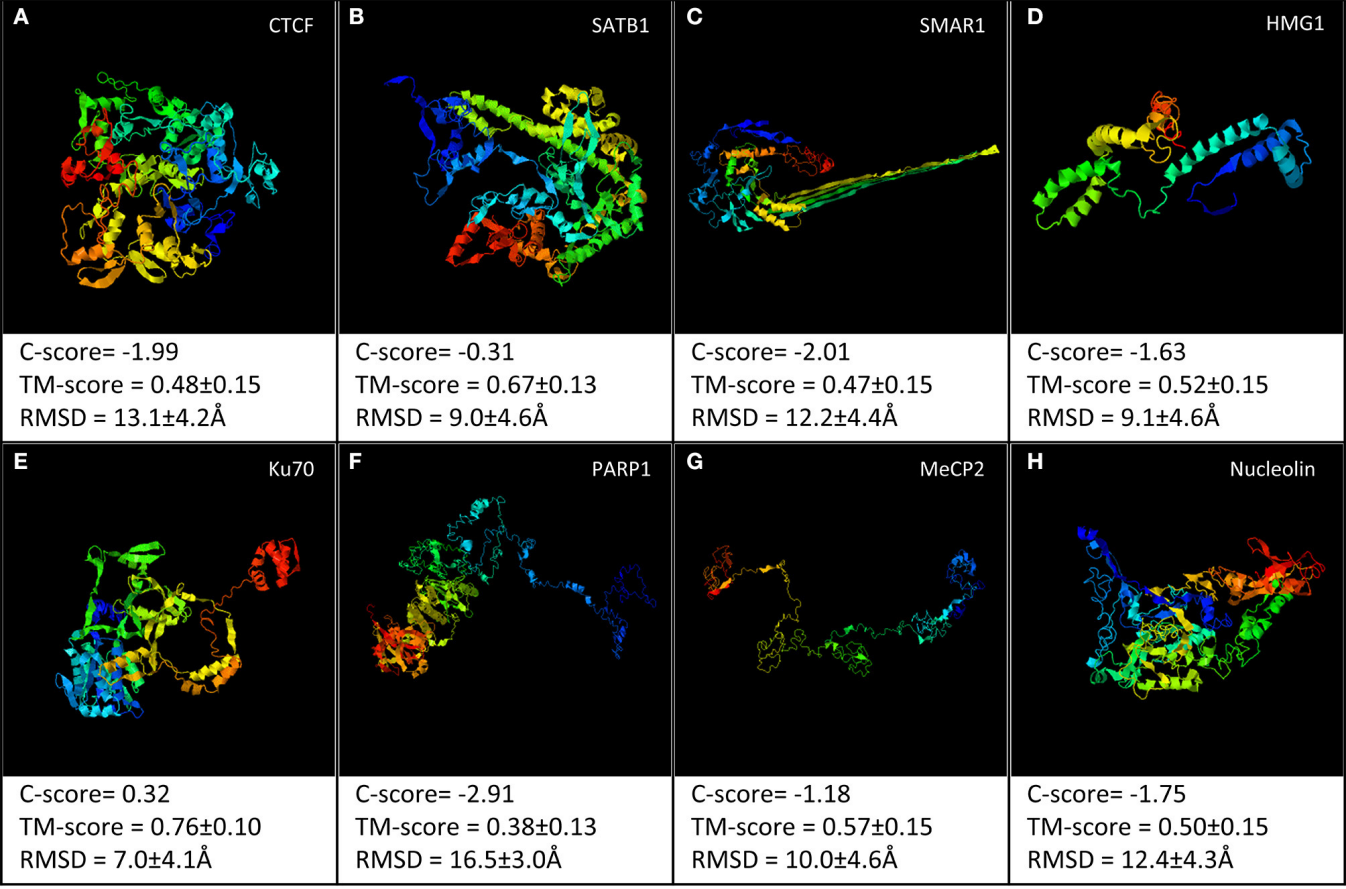
I-TASSER web server was used to predict the structural models for the following proteins: **(A)** CTCF, **(B)** SATB1, **(C)** SMAR1, **(D)** HMG1, **(E)** Ku70, **(F)** PARP1, **(G)** MeCP2, and **(H)** nucleolin. The best model out of the top five predicted models for each has been shown.

**Figure 3:**
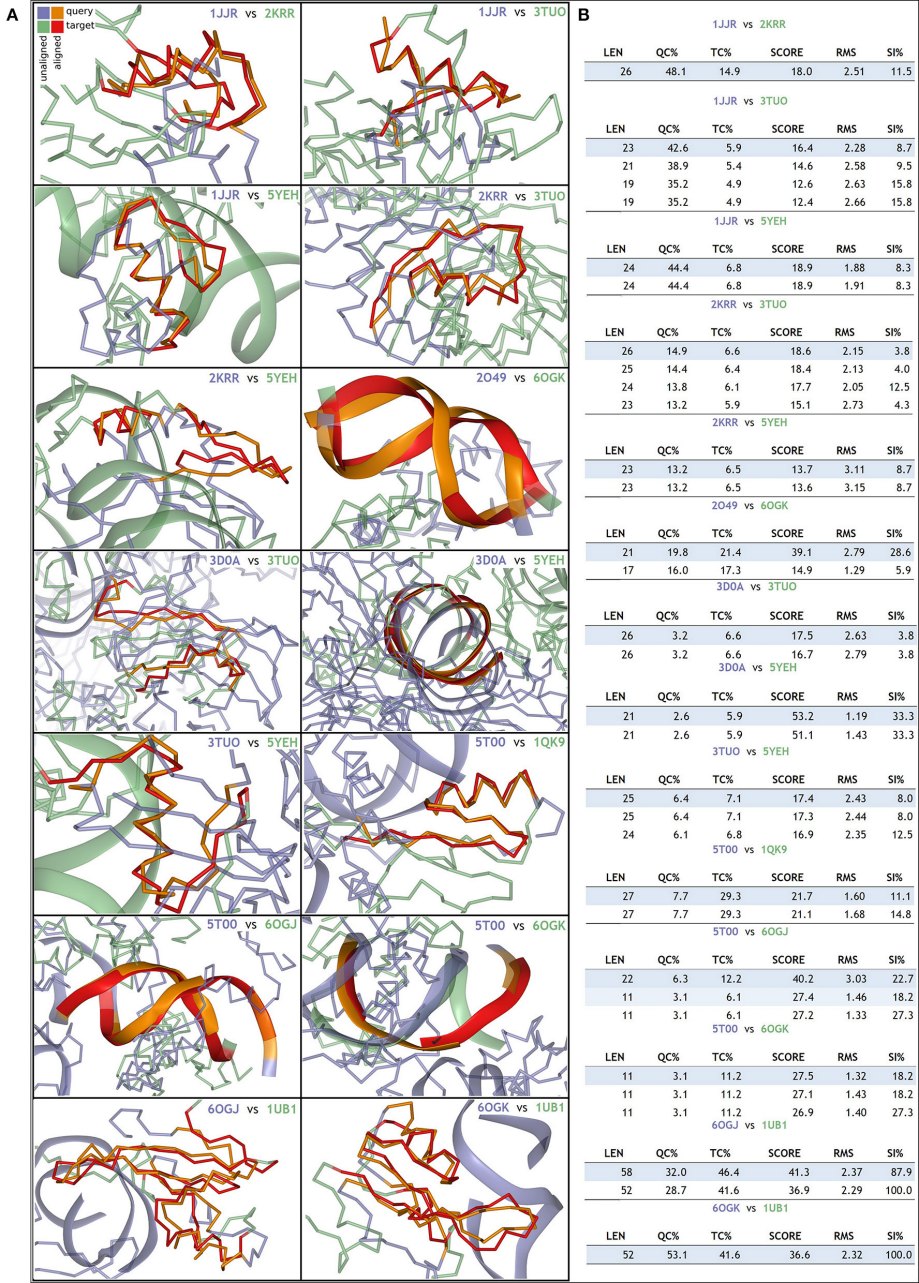
TopMatch tool was used to perform a pair-wise structural alignment between the PDB structures: **(A)** alignment between 1JJR (C-terminal DNA-binding domain of human Ku70) vs. 2KRR (RBD 1,2 domains of human nucleolin), 1JJR vs. 3TUO (N-terminal domain of human SATB1), 1JJR vs. 5YEH (CTCF ZFs4-8-eCBS), 2KRR vs. 3TUO, 2KRR vs. 5YEH, 2O49 (N-terminal CUT domain of SATB1 bound to S/MAR DNA) vs. 6OGK (MeCP2 methyl-binding domain in complex with DNA), 3D0A (Human MUT p53 R249S with second site suppressor mutation H168R, core domain in complex with DNA) vs. 3TUO, 3D0A vs. 5YEH, 3TUO vs. 5YEH, 5T00 (Human CTCF ZnF3-7 in complex with methylated DNA) vs. 1QK9 (MeCP2 DNA-binding domain in complex with methylated DNA), 5T00 vs. 6OGK, 5T00 vs. 6OGJ (MeCP2 methyl-binding domain in complex with DNA), 6OGJ vs. 1UB1 (S/MAR-binding domain of chicken MeCP2), and 6OGK vs. 1UB1. **(B)** Details of the sequence length aligned and the corresponding scores.

It is evident that present structural information does not allow us to conclusively comment on a direct structure–function relationship. Most of the S/MARBPs do not have the crystal structure of entire protein. Rather, each has the structure available for specific domains either alone or in association with some inhibitor or DNA sequence. The X-ray crystallographic structures or in-solution NMR structures only provide a snapshot of a stable conformation out of the possible conformations taken by a protein. Hence, the information is not complete, as the nuclear compartment is highly dynamic in nature. To visualize the structural differences across the different S/MARBPs, we used I-TASSER server (https://zhanglab.ccmb.med.umich.edu/I-TASSER/, Yang and Yang, 2015) to predict the structures of CTCF, SATB1, PARP1, Ku70, MeCP2, SMAR1, nucleolin, and HMG1 based on the sequence information (Figure 2). Although all of them are involved in chromatin looping, they varied in terms of their predicted 3D structures. We used the TopMatch (https://topmatch.services.came.sbg.ac.at/, Wiederstein and Sippl, 2020) web server to assess the structural similarities between the domain structures of the S/MARBPs (viz., CTCF, SATB1, PARP1, Ku70, MeCP2, nucleolin, and MUT p53) using X-ray crystallographic or in-solution NMR structures available at PDB (Figure 3). The following pairs of structures with the respective PDB IDs were used for the analysis: 1JJR (C-terminal DNA-binding domain of human Ku70) vs. 2KRR (RBD 1,2 domains of human nucleolin), 1JJR vs. 3TUO (N-terminal domain of human SATB1), 1JJR vs. 5YEH (Human CTCF ZFs4-8-eCBS), 2KRR vs. 3TUO, 2KRR vs. 5YEH, 3D0A (Human MUT p53 R249S with second site suppressor mutation H168R, core domain in complex with DNA) vs. 3TUO, 3D0A vs. 5YEH, 3TUO vs. 5YEH, 5T00 (Human CTCF ZnF3-7 in complex with methylated DNA) vs. 1QK9 (MeCP2 DNA-binding domain in complex with methylated DNA), 5T00 vs. 6OGK (MeCP2 methyl-binding domain in complex with DNA), 5T00 vs. 6OGJ (MeCP2 methyl-binding domain in complex with DNA), 6OGJ vs. 1UB1 (S/MAR-binding domain of chicken MeCP2), 6OGK vs. 1UB1, and 2O49 (N-terminal CUT domain of SATB1 bound to S/MAR DNA) vs. 6OGK. TopMatch uses several parameters to provide a ranked list of aligned regions. The parameters are as follows: LEN (the alignment length or the number of residues in both the target and query proteins that are structurally equivalent), QC% (query cover depending on the alignment length in query protein and expressed in terms of percentage as QC% = 100 × LEN/Q_n_, where Q_n_ corresponds to the number of residues in the query structure), TC% (target cover dependent on the alignment length in the target protein and expressed in terms of percentage as TC% = 100 × LEN/T_n_, where T_n_ is the number of residues in the target structure), SCORE (a measure of structural similarity; when there is a 100% match between structurally aligned regions of the query and target proteins, the score is equal to length of the aligned regions, with increasing spatial deviation of the aligned amino acid residues, and the score approaches 0), RMS (it is the root mean square error for the superimposition of the structurally aligned regions in Ângstrom calculated using all the structurally comparable C-alpha atoms in proteins), and SI% (Sequence Identity in the structurally aligned regions for both query and target proteins expressed in terms of percentage). In almost all the cases, percentage similarity is quite low, indicated by the low scores. Only alignments between the structures, viz., MeCP2 methyl-binding domain (MBD) and SATB1 CUT domain, both DNA bound and human and chicken MeCP2 MBD bound to DNA displayed higher scores.

**Figure 4:**
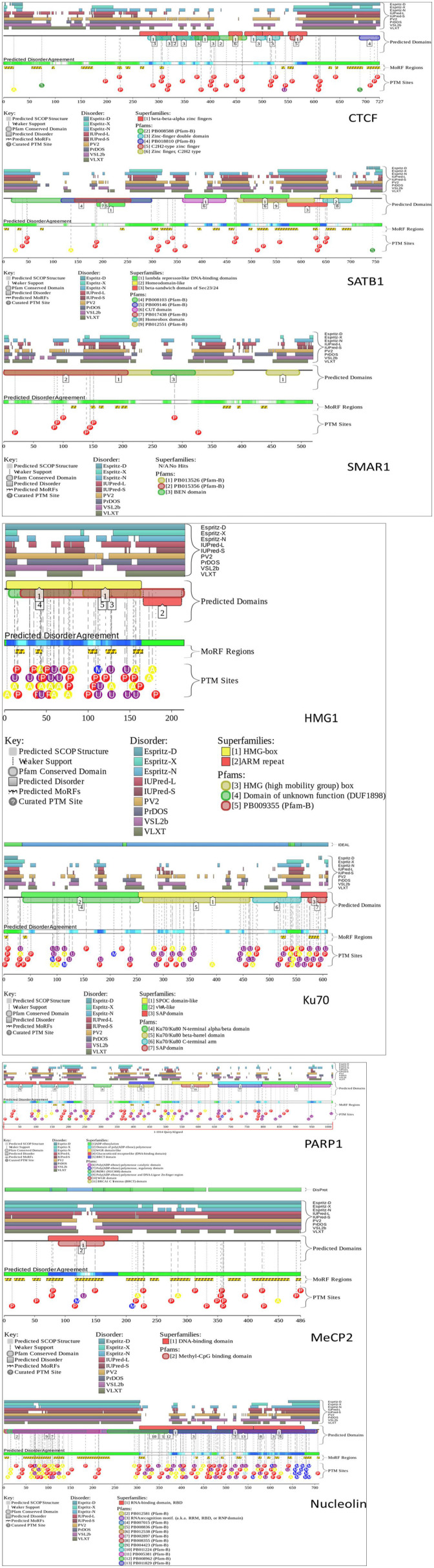
D^2^P^2^ tool was used to assess the intrinsically disordered regions (IDRs) in the following S/MARBPs: CTCF, SATB1, SMAR1, PARP1, MeCP2, nucleolin, HMG1, and Ku70. It uses the following tools for prediction of disordered regions: PONDR VL-XT, PONDR VSL2b, PrDOS, Espritz, PV2, IUPred, and ANCHOR. The pastel-colored blocks (disorder predictions) are aligned and stacked against the polypeptide chain in black. The SCOP (structural classification of proteins) domains are represented by the brightly colored rounded blocks. The agreement level across the different predictors is displayed by color intensity in aligned bar and stacked below the predictions. The yellow blocks having zigzag infills represent the ANCHOR-binding region predictions along with PTM sites predicted by PhosphoSitePlus.

**Figure 5:**
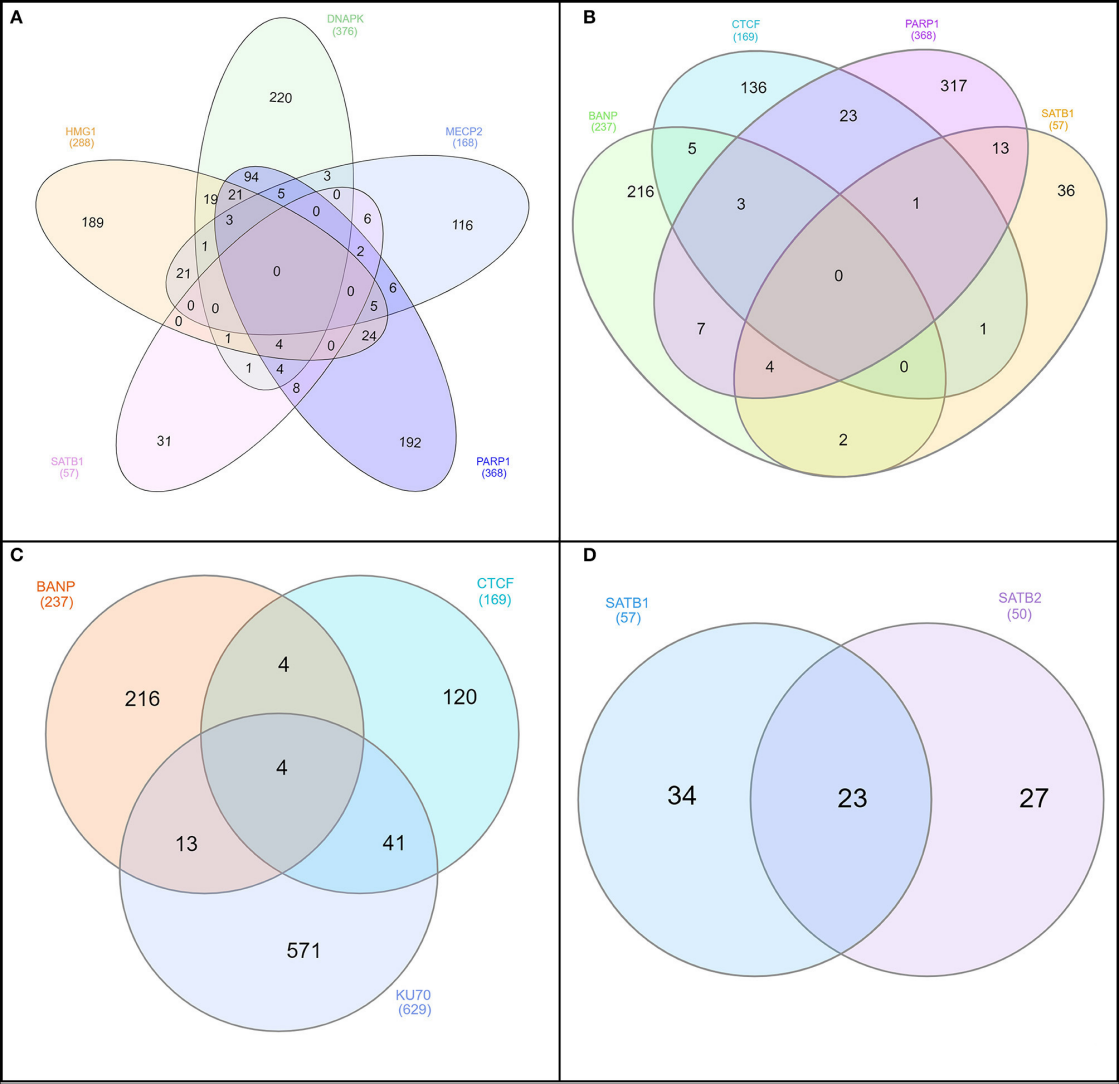
InteractiVenn was used to construct the Venn diagrams to understand similarity of protein–protein interactions (PPIs) between different combinations of S/MARBPs: **(A)** HMG1, DNAPK, MeCP2, SATB1, and PARP1; **(B)** BANP (SMAR1), CTCF, PARP1, and SATB1; **(C)** BANP, CTCF, and Ku70; and **(D)** SATB1 and SATB2. The numerical values represent the number of interacting partners.

Most of the time, the functions of proteins are not correlated with the corresponding 3D structures. Rather, proteins take up different conformations that are inter-convertible depending on the function. Therefore, such proteins have stretches of regions that lack any stable secondary or tertiary structures. These regions are known as intrinsically disordered regions (IDRs). Because these are flexible regions of a protein, they impart a functional advantage over other structurally stable regions, allowing for multiple PPIs (Darling and Uversky, 2018). Interestingly, many of the S/MARBPs have IDRs (Banani et al., 2016; Harmon et al., 2017). These regions of disorder mediate multiple protein interactions and are functionally important. Therefore, we analyzed the IDRs in some of the representative S/MARBPs using the Database of Disordered Protein Prediction (D^2^P^2^, http://d2p2.pro/, Oates et al., 2012) and found that the IDRs also varied across the S/MARBPs (Figure 4). D^2^P^2^ presently includes the tools PONDR VL-XT, PONDR VSL2b, PrDOS, Espritz, PV2, IUPred, and ANCHOR to predict disordered regions that undergo transitions during PPIs. Interestingly, IDRs have several predicted PTM sites (Lieutaud et al., 2016). In a mechanistic view, IDRs are highly dynamic in nature and will allow multiple enzymes to access and post-translationally modify the residues residing in those regions. Human proteins with multiple PTM sites have greater IDRs and behave as hubs/superhubs for in PPI networks. There proteins have more interacting partners and are enriched in protein complexes (Huang et al., 2014). The protein kinases and phosphatases constitute one of the largest gene families in eukaryotes with ~520 kinases and 150 phosphatase coding genes in the human kinome. In humans, greater than two thirds of the total human proteome were found to be phosphorylated (Darling and Uversky, 2018), making phosphorylation as one of the most predominant and important PTMs. We therefore predicted the possible phosphorylations using DisPhos tool (http://www.dabi.temple.edu/disphos/, Iakoucheva et al., 2004) at the IDRs of the S/MARBPs and found multiple sites summarized in Table 1. PTMs at IDRs would be helpful in creating temporarily stabilized regions required for specific PPIs. We next checked for the validated interactome of some selected S/MARBPs using BioGRID (https://thebiogrid.org/, Stark et al., 2006) and generated Venn diagrams using InteractiVenn (http://www.interactivenn.net/, Heberle et al., 2015) to understand the similarities and dissimilarities in terms of PPIs (Figure 5). In most of the cases, the overlaps are low. SATB1 and SATB2 belong to the same family of proteins, yet they have roughly 50 percent interactions in common. A list of the possible PTMs using the web servers Kinexus:PhosphoNET (http://www.phosphonet.ca/) and NetPhos 3.1 (http://www.cbs.dtu.dk/services/NetPhos/, Blom et al., 1999, 2004) for phosphorylation, PMeS (http://bioinfo.ncu.edu.cn/inquiries_PMeS.aspx, Shi et al., 2012) and MethylSight (https://methylsight.cu-bic.ca/predictor, Biggar et al., 2020) for lysine methylation, GPS-PAIL 2.0 (http://pail.biocuckoo.org/online.php, Li et al., 2006; Deng et al., 2016) for lysine acetylation, GPS SUMO 2.0 (http://sumosp.biocuckoo.org/online.php, Ren et al., 2009; Zhao et al., 2014) for SUMOylation, GPS-SNO 1.0 (http://sno.biocuckoo.org/, Xue et al., 2010) for *S*-nitrosylation, and GPS-Palm (http://gpspalm.biocuckoo.cn/, Ren et al., 2008; Ning et al., 2020) for palmitoylation have been summarized in Table 2. Thus, all the information supports the fact that PTMs will be crucial in altering PPIs of S/MARBPs, thereby orchestrating their gene regulatory functions. Hence, in this review, we delve into the current understanding of S/MARBP PTMs in regulating DNA binding, gene expression, and their disease implications.

**Table 1:** Predicted phosphorylation sites within the IDRs of S/MARBPs.

**Protein**	**Position**	**Score**	**Protein**	**Position**	**Score**
CTCF	T24	0.89	Ku70	S2	0.89
CTCF	S190	0.81	Ku70	S6	0.83
CTCF	Y197	0.91	Ku70	T10	0.85
CTCF	S210	0.91	Ku70	S27	0.85
CTCF	S594	0.95	Ku70	S37	0.80
CTCF	S604	0.99	Ku70	S184	0.85
CTCF	S609	0.98	Ku70	S257	0.95
CTCF	S610	0.99	Ku70	S550	0.84
CTCF	S612	0.98	Ku70	S552	0.91
CTCF	T642	0.91	Ku70	S560	0.86
SATB1	S47	0.79	PARP1	S4	0.85
SATB1	S289	0.73	PARP1	S16	0.86
SATB1	S292	0.72	PARP1	S20	0.86
SATB1	S451	0.89	PARP1	S224	0.97
SATB1	S465	0.73	PARP1	S232	0.96
SATB1	S469	0.83	PARP1	S375	0.87
SATB1	T620	0.74	PARP1	S499	0.90
SATB1	T630	0.76	PARP1	S504	0.88
SATB1	S633	0.86	PARP1	S782	0.88
SATB1	S637	0.90	PARP1	S808	0.88
SMAR1	S179	0.75	MeCP2	S178	0.98
SMAR1	S181	0.80	MeCP2	S341	0.98
SMAR1	S184	0.69	MeCP2	S346	0.99
SMAR1	S186	0.61	MeCP2	S349	0.99
SMAR1	T337	0.62	MeCP2	S350	0.99
SMAR1	S340	0.64	MeCP2	S355	1.00
SMAR1	S341	0.60	MeCP2	S356	1.00
SMAR1	S346	0.77	MeCP2	S357	0.99
SMAR1	S351	0.78	MeCP2	S395	0.99
SMAR1	S357	0.60	MeCP2	S396	0.99
HMG1	S14	0.64	Nucleolin	S28	1.00
HMG1	S15	0.58	Nucleolin	S34	1.00
HMG1	S35	1.00	Nucleolin	S41	0.97
HMG1	S39	0.53	Nucleolin	S42	0.96
HMG1	S46	0.81	Nucleolin	S145	1.00
HMG1	S53	0.88	Nucleolin	S153	1.00
HMG1	Y78	0.63	Nucleolin	S184	1.00
HMG1	S100	0.83	Nucleolin	S206	0.99
HMG1	S181	0.99	Nucleolin	S386	0.89
			Nucleolin	S563	0.89

**Table 2:** Predicted post-translational modifications of the different human S/MARBPs.

**Protein**	**Phosphorylation**	**Acetylation**	**Methylation**	**Sumoylation**	**Palmitoylation**	**Nitrosylation**
CTCF	T129	K206	K278	K74	C268	C38
	S130	K490	K320	K605	C271	
	T204	K514	K371	K606	C528	
	S224	K592	K487	K689	C560	
	S287	K593	K490			
	T391	K595	K493			
	T417	K599	K496			
	S488	K601	K543			
	S604	K605	K596			
	S610	K606	K652			
SATB1	T188	K11	K11	K175		C209
	S212	K29	K24	K535		
	S255	K51	K29	K744		
	S497	K475	K42	K762		
	S539	K486	K44			
	S637	K523	K70			
	S652	K644	K136			
	Y687	K705	K427			
	S710	K762	K642			
	S742		K735			
SMAR1	S76	K80	K59	K133	C315	C231
	S139	K275	K296		C435	
	S256	K278	K308			
	S327	K279	K314			
	S345		K316			
	T352		K320			
	S375		K322			
	Y379		K324			
	S476		K443			
	S479					
HMG1	S14	K3	K3	K82		
	T22	K7	K8	K114		
	S42	K12	K110	K167		
	S53	K173	K147	K177		
	T85	K177	K150	K180		
	S100	K180	K152	K182		
	S181	K182	K163	K183		
		K183		K184		
		K184		K185		
		K185				
Ku70	S78	K164	K92	K9		C66
	T90	K182	K114	K510		C389
	S162	K249	K207	K556		
	S180	K539	K258	K596		
	T251	K542	K399			
	S257	K544	K575			
	S477	K553	K591			
	S520	K556	K595			
	S560	K575	K596			
	T572	K605	K605			
PARP1	S20	K15	K10	K203		C24
	S63	K209	K15	K249		C298
	S123	K222	K78	K486		C311
	S140	K226	K207	K512		
	Y309	K230	K505	K798		
	T432	K233	K506	K838		
	S468	K236	K522			
	S785	K418	K524			
	S808	K505	K591			
	S947	K949	K878			
MeCP2	S86	K42	K12	K32		
	T105	K175	K22	K42		
	S166	K180	K24	K61		
	S178	K254	K36	K135		
	S204	K271	K111	K363		
	T241	K345	K119			
	S332	K347	K186			
	S350	K352	K190			
	S395	K364	K210			
	S486	K377	K254			
Nucleolin	S34	K6	K109	K15		
	S60	K132	K656	K135		
	S145	K135	K660	K138		
	S153	K138	K666	K141		
	S206	K141	K673	K142		
	S386	K142	K687	K377		
	Y495	K217	K701	K437		
	T501	K219	K705	K648		
	T583	K228	K706	K708		
	S619	K708	K708			

## Post-Translational Modification as Regulators of Scaffold/Matrix Attachment Region-Binding Proteins

### Phosphorylation

**Figure 6:**
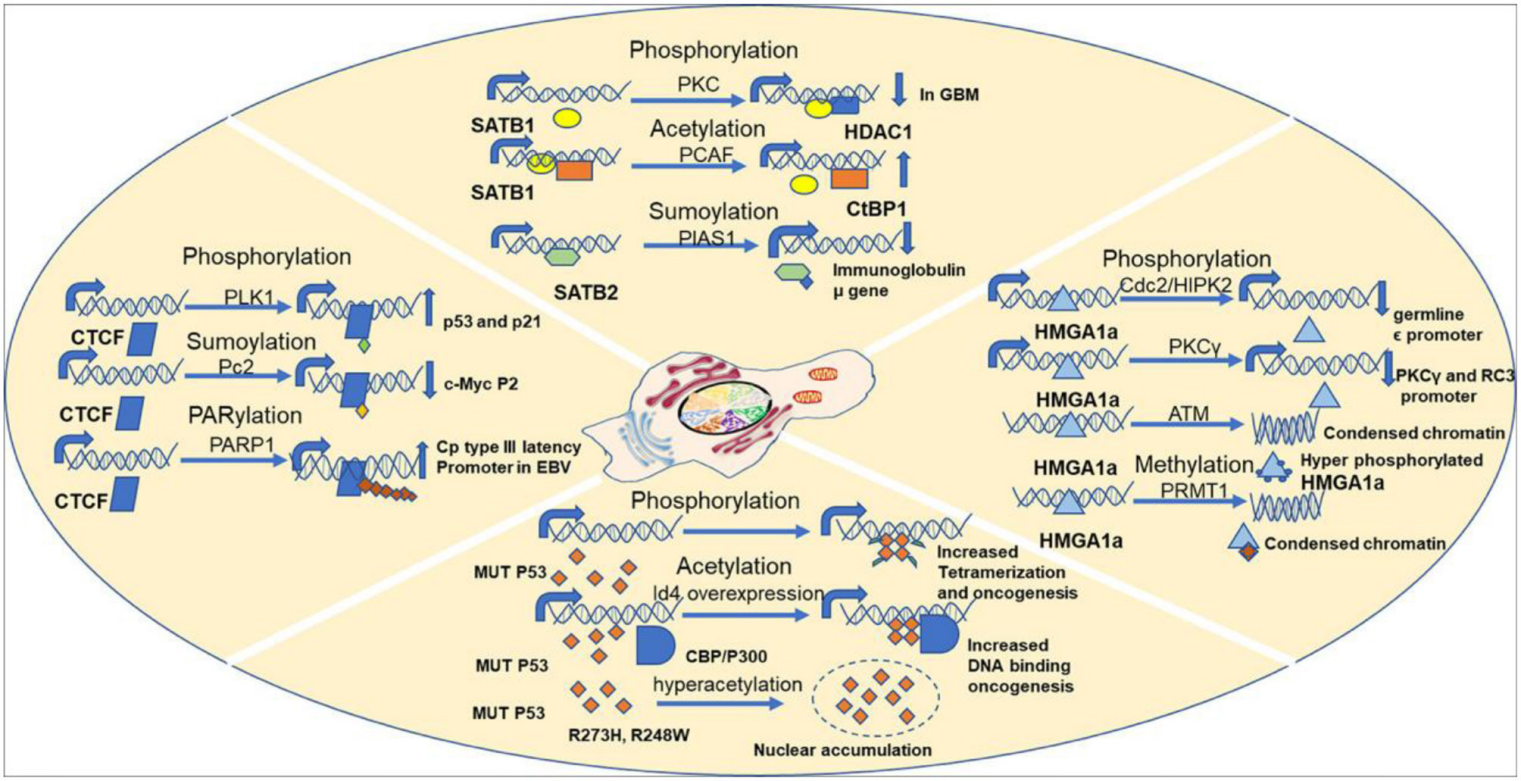
Examples showing importance of PTMs in regulating S/MARBP functions.

Protein phosphorylation is one of the most predominant and well-studied PTMs that mediate functional changes. Protein kinases phosphorylate corresponding protein substrates at specific positions, leading to conformational changes that alter the function in turn. Studies have confirmed overexpression of both phospho SATB1 and protein kinase C (PKC) in glioblastoma, a deadly form of cancer that originates in the brain (Figure 6). Interestingly, there was a reduction in both mRNA and protein levels of SATB1 in cancer. But an increased phosphorylation of SATB1 by PKC caused enhanced association with histone deacetylase (HDAC) 1, leading to altered transcription of genes, which contributed to the aggressive behavior of this cancer. Although SATB1 expression was reduced at both the mRNA and protein levels across increasing grades of cancer, phosphorylation was increased, leading to functional changes (Han et al., 2013). The cut homeodomain proteins from *Drosophila* and mammals have additional three DNA-binding domains apart from the homeodomain called cut repeats. Experiments using mammalian nuclear extracts have shown that PKC can phosphorylate cut repeats, causing an inhibition of its DNA-binding activity. Site-directed mutagenesis studies have shown that murine cut is phosphorylated by PKC at Thr415, Thr804, and Ser987 in cut repeats 1–3, respectively (Coqueret et al., 1996). Similar studies using mammalian nuclear extracts have demonstrated that Casein kinase II can also phosphorylate cut repeats, leading to decrease in DNA-binding activity. The murine cut phosphorylation sites are Ser400, Ser789, and Ser972, respectively, in cut repeats 1, 2, and 3 (Coqueret et al., 1998). The DNA-binding activity of p110 Cux/CDP was found to be downregulated by cyclin A/cdk1-mediated phosphorylation in the G_2_ phase of cell cycle. *In vitro* studies confirmed the phosphorylation sites to be Ser1237 and Ser1270 (Santaguida et al., 2001). Lamins are the major building blocks of nuclear skeleton. Lamins bind to S/MARs and help in chromatin organization. Lamins undergo several PTMs out of which phosphorylation is the only easily reversible and common modification. The DNA-binding motif resides in the central (rod) domain (Glass et al., 1993; Zhao et al., 1996) and in the tail domain (Stierlé et al., 2003). Reports suggest that lamins mainly bind DNA via tail domain, which harbor several kinase sites. For example, in *Xenopus, in vitro* nuclear assembly system pseudophosphorylation of Ser37 of fly lamin C led to increase in mobility and solubility with reduction in polymerization and chromatin binding (Zaremba-Czogalla et al., 2012). Studies have shown that PKCα phosphorylates lamin B in HL60 cells, leading to its proteolysis prior to DNA fragmentation during apoptosis (Shimizu et al., 1998). Inhibition of nuclear import of lamins would also alter chromatin organization, in turn changing the gene expression pattern. The GRASS404 sequence of mammalian lamin C (Eggert et al., 1993; Haas and Jost, 1993; Leukel and Jost, 1995) and Ser410/411 of chicken lamin B2 (Hennekes et al., 1993) are both targets of PKC. These phosphorylations are essential for nuclear import of lamins. MeCP2 is another multifunctional S/MARBP. Its importance is highlighted by the finding that mutations in this protein cause Rett syndrome, a neurodevelopmental disorder. It is yet not fully understood how it regulates functions of neuronal cells. The entire spectrum of PTMs with the corresponding functions for this protein is yet not explored. However, mutational analysis has shown that Ser80 phosphorylation modulates its association with the chromatin at promoters of some genes in resting neuron, while calcium influx causes dephosphorylation, leading to its dissociation from chromatin (Tao et al., 2009). Membrane depolarization of neurons led to phosphorylation of Ser421 in MeCP2, leading to activation of brain-derived neurotrophic factor (bdnf) promoter in neuronal cells. This occurs due to reduced binding of phosphorylated MeCP2 to the promoter (Zhou et al., 2006). Another interesting study highlighted the role of Ser421 phosphorylated (pS421) MeCP2 in mouse neuronal development and function. To understand its genome-wide role, the authors used pS421 antiserum to perform ChIP analysis after neuronal stimulation using KCl depolarization. The data demonstrated that pS421 is evenly distributed across the MeCP2 proteins bound to the genome. An estimation revealed that 10–30% of total MeCP2 was phosphorylated in response. This study showed that pS421 MeCP2 is quite common in occurrence and probably has a more global regulatory role in modulating the neuronal chromatin in response to sensory stimuli. Upon loss of pS421 MeCP2, there were observable defects in experience induced neuronal development. Ser423 is the corresponding site in human MeCP2 and may be similarly phosphorylated and warrants further investigation (Cohen et al., 2011). MFP1 is a conserved plant S/MARBP that is found in the stroma of thylakoids and in the nucleus. The homologous protein in *Allium cepa*, AcMFP1, was reported to be phosphorylated by CKII in a cell cycle-dependent manner with moderate phosphorylation at G_2_ phase. This weakens its interaction with the nuclear matrix (Samaniego et al., 2006). Similar studies have shown that chloroplast-localized MFP1 is phosphorylated in *Nicotiana tabacum* L. (tobacco) by CKII, leading to decreased association with the chloroplast nucleoid (Meier et al., 1996).

There exist three families of HMG proteins, viz., HMGA, HMGB, and HMGN. They are DNA-binding proteins that can regulate chromatin organization and gene transcription. Like histone proteins, HMG proteins also undergo several PTMs that regulate their nuclear functions. HMGA proteins are hardly detectable in human adult tissues. However, HMGA proteins were found to be overexpressed in cancers. Earlier studies have shown that HMGA1a protein, a member of HMGA family, is phosphorylated by cdc2 at Thr52 and Thr77 *in vitro* and in metaphase arrested cells, leading to reduction in DNA-binding ability (Nissen et al., 1991; Reeves et al., 1991). Ser35 is another site apart from Thr52 and Thr77 that is phosphorylated by cdc2, leading to similar effect (Zhang and Wang, 2007) (Figure 6). Homeodomain-interacting protein kinase 2 (HIPK2) also phosphorylates HMGA1a at Ser35, Thr52, and Thr77 in a cell cycle-dependent manner, leading to lowering of DNA-binding affinity (Figure 6). It reduced occupancy at the human germ line ε promoter (Zhang and Wang, 2007). Similarly, PKC phosphorylates HMGA1a at Ser43 and Ser63, which attenuates binding to PKCγ and neurogranin/RC3 gene promoters (Xiao et al., 2000) (Figure 6). Recent studies have demonstrated that HMGA1 proteins are important players of DNA damage signaling pathway. HMGA1 proteins are downstream targets of ataxia telangiectasia mutated (ATM) kinase pathway. Hyperphosphorylaton of HMGA1a was observed in early apoptotic leukemia cells (U937, K562, HL60, and NB4), which could be associated with displacement from chromatin, while the dephosphorylated form was seen during formation of apoptotic bodies containing highly condensed chromatin (Diana et al., 2001) (Figure 6). ATM phosphorylates HMGA1b at Ser87 within SQ motif (Pentimalli et al., 2008). In eukaryotic cells, major steps of ribosomal biogenesis occur in the nucleolus. Ribosomal RNA gene transcription occurs actively in the nucleolus. Studies using adult bovine arterial endothelial cells have shown that in confluent cells, the rate of transcription of rDNA genes falls to about 5% of that in growing cells. Further analysis revealed that this regulation is mediated via phosphorylation of nucleolin, an S/MARBP by protein kinase NII. Upon phosphorylation by protein kinase NII, nucleolin activity is enhanced, leading to increased rDNA transcription (Bclenguer et al., 1989). The neural restrictive silencer factor (REST) is a tumor suppressor gene that when mutated can cause epithelial transformation. Malignant cells overexpress several kinases that promote cellular proliferation and survival. With the use of a panel of cancerous cell lines, it was demonstrated that REST and nucleolin had overlapping binding sites in the promoter of cd59 gene. The gene cd59 is a membrane protein that is frequently overexpressed in cancer. Its primary function is to prevent the formation of membrane attack complex. However, it was phosphorylated nucleolin that competed with REST at the promoter. PI3K and PKCξ kinases lead to REST degradation and phosphorylate nucleolin resulting in REST displacement from the promoter. Because of this, cd59 expression is upregulated (Tediose et al., 2010). DNA repair is essential for maintenance of genomic integrity and cellular survival. SMAR1 has been reported to be phosphorylated by ATM kinase at Ser370, which helps in recruitment of deacetylated Ku70 at DNA double-stranded breaks (DSBs) in the cell. This ensures timely repair of DSBs, thus ensuring genomic integrity (Chaudhary et al., 2014). While WT p53 is the guardian of genome, MUT P53 is being considered the guardian of cancer genome (Mantovani et al., 2019). Interestingly, MUT p53 is phosphorylated at several sites like the WT p53, although the pattern differs. This imparts oncogenic functions to it. Almost 20 years back in 1993, a pioneering study demonstrated that even under unstressed conditions, phosphorylation status of certain residues differed in MUT p53 compared with WT p53. In tumor cells harboring MUT p53s, phosphorylation at Ser15 was reduced but increased at Ser392. However, at Ser9, there was no change (Ullrich et al., 1993). It was also confirmed that MUT p53 could be phosphorylated at Thr81, Ser392, and Ser15 *in vivo* in tumors (Minamoto et al., 2001). Phosphorylation of the MUT p53 is expected to alter the conformation, thereby altering its oncogenic functions. This is substantiated by several studies. Mutations at Ser15 and 46 in the N-terminal region lead to variability in radiosensitivity of lung cancer cells (Okaichi et al., 2011). The N-terminus is phosphorylated by JNK (Zerbini et al., 2005), and the C-terminus is phosphorylated by PLK2 (Valenti et al., 2011), both of which lead to enhanced oncogenic properties. Ser392 is one of the few well-studied sites that undergo PTM in MUT p53. Located within the C-terminus of MUT p53, it is frequently found to be hyperphosphorylated in tumors having Arg248Trp and Arg273His hot spot mutations (Ullrich et al., 1993; Minamoto et al., 2001; Warnock et al., 2011). Almost 60% of human transitional cell carcinomas harboring MUT p53 missense mutations have constitutive phosphorylation of Ser392 (Furihata et al., 2002). Other studies have shown that higher frequency of hyperphosphorylated Ser392 correlates with poor prognosis in a variety of cancers like skin tumors and esophageal squamous cell carcinomas (Matsumoto et al., 2004a,b; Bar et al., 2009). However, in breast tumors, Ser392 shows reduced phosphorylation (Yap et al., 2004). Thus, it is evident that there remain tissue-specific oncogenic roles of MUT p53 based on phosphorylation status. Ser392 phosphorylation is expected to stabilize MUT p53 tetramer, thus promoting DNA binding and other oncogenic properties (Bode and Dong, 2004) (Figure 6). The ZF CCCTC-binding protein or CTCF is a multifunctional protein that participates in numerous unrelated functions including fine-tuning of developmentally regulated genes and imprinted loci, negative or positive regulation of transcription, and inactivation of X-chromosome. Interestingly, CTCF teams up with different partners like RNA polymerase II, Yy1, and PARP1 to perform various functions (Zlatanova and Caiafa, 2009), raising the question of PTMs in mediating that. A recent study aimed at CTCF mass spectrometry (MS) and detected a novel phosphorylation at Ser224 by Polo-1-like kinase (PLK1) (Figure 6). This post-translationally modified CTCF is found to be chromatin associated and at a subset of CTCF-binding regions. This accumulates in the G2/M phase of cell cycle. Experiments using mouse embryonic stem cells have shown that the Ser224E that mimics constitutive phosphorylation does not have any impact on chromatin architecture and ploidy. However, it affects the gene expression of several hundreds of genes including important ones like p21 and p53. On the other hand, phosphorylation-defective mutant Ser224A does not seem to have any effect (Del Rosario et al., 2019). This fact that phosphorylation-defective mutant does not have an impact indicates that there are other such factors that have functional redundancy. A recent study identified CTCF as a novel target of large tumor suppressor kinase 1 (LATS1), a negative regulator of YAP1 protein in Hippo signaling pathway. The Hippo–LATS pathway is extensively involved in central processes like tissue homeostasis, organ size maintenance, cell proliferation, apoptosis, and tumorigenesis. Under cellular stress, LATS1 gets activated, phosphorylates YAP, and promotes its sequestration in the cytoplasm via 14-3-3 protein binding. CTCF contains 11-ZF motifs out of which the central 4- to 7-ZF motifs form the DNA-binding core. Activated LATS1 phosphorylated CTCF ZFs at Thr374 and Ser402 under cellular stress. Genome-wide ChIP sequencing of CTCF under cellular stress like serum starvation and glucose starvation revealed that LATS1-phosphorylated CTCF was dissociated from a small subset of binding sites that were mainly enriched for anchors of chromatin domains containing target genes of YAP. This resulted in disruption of local chromatin domains and reduced expression of YAP target genes located inside those. This study therefore uncovered the “signal-responsive plasticity of 3D genome architecture” and pinpoints CTCF ZF phosphorylation as the mechanism. This study showed that cellular stress disrupts CTCF-mediated chromatin looping selectively at YAP target genes (Luo et al., 2020). Thus, it is evident through these numerous studies that phosphorylation is an important PTM that can directly alter the DNA-binding ability of S/MARBPs, thereby fine-tuning gene expression and chromatin organization for maintenance of cellular homeostasis.

### Acetylation

Acetylation is another major PTM that occurs in cells that regulate cellular homeostasis. Histones are known to be acetylated at the lysine residues in their tails. However, there are ample reports of non-histone protein acetylation. S/MARBPs are also reported to be acetylated, which in turn govern their functions. SATB1 is a global regulator of gene expression. It can interact with different coactivator or corepressor complexes in a tissue- and gene-specific manner. SATB1 can form a repressor complex with C-terminal-binding protein 1 (CtBP1) *in vivo* in mammalian cells. Acetylation of SATB1 by p300/CBP-associated factor (PCAF) at Lys136 in PDZ domain led to disruption of its interaction with CtBP1 resulting in upregulation of expression of its target genes (Purbey et al., 2009) (Figure 6). Interferon-β (IFN-β) is important in mediating cellular response to viral infections. A tightly regulated assembly and disassembly of higher-order nucleoprotein complex called enhanceosome control expression of this gene. HMGI(Y) is an architectural component required for enhanceosome formation and can be regulated by acetylation. A study has shown that acetylation at Lys71 by PCAF/GCN5 activates transcription by stabilizing the enhanceosome, while acetylation at Lys65 by CREB-binding protein (CBP) destabilizes the complex resulting in downregulation (Munshi et al., 2001). Another study identified acetylation of CDP/cut in third cut repeat (C3) and homeodomain (HD) regions by PCAF. This again resulted in reduced DNA-binding ability and thus downregulation of target gene expression (Li et al., 2000). Ku70 is an S/MARBP that was initially described as an autoantigen. It is heavily involved in DNA repair pathway. Neuroblastoma is a cancer that occurs in children. It originates in the neural stem cells. Interestingly, aggressive neuroblastoma cells (N) were killed by ionizing radiation, while the less aggressive stromal type (S) was much less susceptible to it. Further investigation solved the puzzle. An increased Ku70 acetylation by CBP in the N type compared with the S type of cells reduced the DNA-binding ability of Ku70, thereby affecting DNA repair mechanism (Subramanian et al., 2013). MeCP2 protein gets methylated at Lys464 by P300 in cultured mouse cortical neurons. Acetylation enhances the DNA-binding ability at bdnf exon 4 as evidenced by chromatin immunoprecipitation experiments using fresh hippocampal tissue. The enhanced binding leads to decrease in transcription, thereby reducing bdnf at both mRNA and protein levels. The deacetylation is mediated by SIRT1 as evident from studies done in mice lacking a functional SIRT1 (Zocchi and Sassone-Corsi, 2012). WT p53 is already known to be post-translationally modified, but interestingly, MUT p53 is also modified. Although majority of acetylation sites are not altered in tumors, Lys120 and Lys164 in the DNA-binding domain of WT p53 are often found to be mutated in several tumors. This suggests important, independent tumor suppressive functions of these two PTMs (Tang et al., 2008). MUT p53 also gets acetylated at several residues in different cancers and, in the absence of stress Arg273His and Arg248Trp, has found to be hyperacetylated at Lys382, Lys373, and Lys320 (Minamoto et al., 2001). These might help in nuclear accumulation of MUT p53 (Figure 6). Almost one third of prostate cancers harbor MUT p53s, making the mechanistic insights into its regulation important. One such study has identified inhibitor of differentiation 4 (Id4) as an important factor regulating function of MUT p53 in prostate cancer. Overexpression of Id4 in DU145 cell line led to increased MUT p53 acetylation at Lys373 mediated by CBP/P300 and promoted its DNA-binding ability (Knowell et al., 2013) (Figure 6). The exact functions of acetylation on both WT p53 and MUT p53 are unclear, as Lys serves as the site for various other modifications like neddylation, methylation, and ubiquitylation. In one of the studies, it has been shown that acetylation of MUT p53 regulates metabolic activities and survival of cancer cells. Glucose restriction leads to acetylation in the C-terminal end of MUT p53, which did not happen in WT p53. This was validated using several cell lines like MDA-MB-231 (Arg280Lys), T47D (Lys194Phe), and PANC1 (Arg280Thr). Using a C-terminal acetylation-mimicking mutant of p53 (Gly245Ala-K6Q), authors established a connection between C-terminal acetylation and autophagy (Rodriguez et al., 2012). This is interesting, as autophagy is a double-edged sword and can be useful for tumor growth when hypoxia sets in due to increase in tumor size. It can thus provide food for those cells in the heterogeneous population, which are more genetically fit to survive and metastasize. Another study revealed that use of HDAC inhibitors or acetylation by PCAF restored partial WT p53 DNA-binding ability of two conformation mutants Arg175His and Gly245Ala (Perez et al., 2010). Therefore, these studies make it evident that acetylation plays an important role in modulating the DNA-binding ability of MUT p53 and regulates several oncogenic functions.

### SUMOylation

SATB2 is a novel S/MARBP that binds to the immunoglobulin μ locus S/MARs and regulates transcription in Pre-B cells. SATB2 is SUMOylated at Lys233 and Lys350 by the SUMO E3 ligase PIAS1 that downregulates the DNA-binding activity (Figure 6). SATB2 Lys233/Lys350 double mutants are associated with immunoglobulin μ gene with five times greater efficiency than the WT form. Therefore, SUMOylation acts to orchestrate SATB2-mediated gene regulation (Dobreva et al., 2003). SAF-B1 is SUMO-1 tagged via PIAS1. This SUMOylation is important for RNA polymerase II association at the promoters of genes encoding ribosomal proteins and translation factors. Therefore, at the promoters of the above-mentioned genes, SUMO1 tagged as SAFB1 acts as a transcriptional activator (Liu et al., 2015). Interestingly, SAFB1 is modified at Lys231 and Lys294 via SUMO1 and SUMO2/3, respectively. These modifications are essential for the corepressor activity. Mutation of SUMOylation site leads to decreased interaction with HDAC3, thus reducing its potential as a transcriptional repressor (Garee et al., 2011). Ku70 is SUMOylated in both yeast and human. Ku70 is involved in both telomeric maintenance and DNA repair mechanism. In budding yeast, the C-terminal tail of Ku70 is SUMOylated on a cluster of five lysine residues, viz., Lys588, Lys591, Lys592, Lys596, and Lys597, which enhances DNA-binding affinity. Mutating the residues compromises both DNA repair and telomere length (Hang et al., 2014). MeCP2 is reported to be SUMOylated at Lys223, which is essential for its transcriptional repression activity. This modification is required for recruiting HDAC1/2 complexes. In rats, abrogating the Lys223 site leads to poor development of hippocampal synapses. Thus, it is crucial for modulating synaptic development in the central nervous system (Cheng et al., 2014). CTCF plays diverse roles and usually follows the divide and rule policy to create diverse impacts on target genes. CTCF has been found to be SUMOylated by SUMO 1, 2, and 3. Two major sites of SUMOylation are in the NH2 terminal domain and –COOH terminal domain. Polycomb 2 (Pc2) protein, a member of Polycomb group, is likely the E3 ligase. CTCF localizes with Pc2 in Polycomb nuclear bodies. This modification of CTCF is important for repressing c-myc P2 promoter (Figure 6). While SUMOylation is required for its repressive role, CTCF mutants defective for SUMOylation indicate only 2-fold change in gene expression, raising the possibility of other PTMs regulating its repressive functions. Interestingly, mutation of the phosphorylation sites in the C-terminal domain of the protein to non-phosphorylatable amino acids leads to increase in CTCF repressive activity. This indicates that a competition might exist between SUMOylation and phosphorylation to regulate CTCF activity reciprocally. Pc2 mediates SUMOylation of CTCF by SUMO 2 and 3. Overexpression of Pc2 led to decrease in CTCF modified by SUMO 1, leading to the speculation that Pc2 might SUMOylate other protein targets by SUMO 1 (MacPherson et al., 2009). Another study using human corneal epithelial cells report de-SUMOylation of CTCF under hypoxic and stress conditions. Upon hypoxia induction, CTCF was de-SUMOylated at lysine 74 and 689. Overexpression of Sentrin-specific protease 1 (SENP1) led to increase in 130-kDa form of CTCF; however, knockdown did not rescue the hypoxia-induced de-SUMOylation, making it evident that there must be other such de-SUMOylating enzymes, which act on CTCF under hypoxic condition. No change was seen in terms of CTCF phosphorylation. Interestingly, and contrary to the previous study, PAX6 was found to be suppressed upon CTCF de-SUMOylation. This is substantiated by the fact that CTCF knockdown led to enhanced expression of PAX6 (Wang et al., 2012).

### Methylation

Non-histone methylation is a lately identified PTM that has elusive roles in regulating protein functions. Several S/MARBPs have been reported to be modified by methylation at either lysine or arginine residues. HMGA1a was found to be methylated in human leukemia cells, rat thyroid tumor cells, and human prostrate tumor. Interestingly, the methylation level was increased during apoptosis. Arg25 within the first AT hook was methylated. Both mono-methylation and di-methylation at this site were detected (Sgarra et al., 2003). Protein arginine methyltransferase 1 (PRMT1) methylated this site *in vivo* (Zou et al., 2007). This methylation is probably involved in chromatin remodeling and heterochromatin formation seen during apoptosis (Figure 6). HMGB1 isolated from neutrophils is reported to be methylated at Lys42, which causes a conformational change, leading to weakening of DNA-binding activity. This in turn altered its subcellular localization from nucleus to cytoplasm (Ito et al., 2007). WT p53 has been shown to be mono-methylated or di-methylated at four different sites in its C-terminal domain by at least six different lysine methyltransferases. This facilitates binding by PHF20, TIP60, 53BP1, and L3MBTL1. All these protein binders help in modifying p53 activity. For example, SMYD2 monomethylates WT p53 at Lys370, leading to a repression of its activity, while methylation of Lys372 by SET7/9 leads to stabilization of chromatin-bound WT p53 (West and Gozani, 2011; Scott et al., 2012). Therefore, methylation of WT p53 modulates its activity by fine-tuning localization, stability, and DNA-binding ability and so might also regulate MUT p53. Hence, functional implications of this PTM are emerging, but it is still in its infancy.

### PARylation

Poly-ADP ribosylation has lately received much attention in the field of PTMs. This process is catalyzed by PARP. In humans, there are ~18 such PARPs. This group of enzymes catalyzes the transfer of poly (ADP-ribose) to the target proteins. Although some of the isoforms like PARP1 and PARP2 play a role in DNA damage repair, recent studies show their involvement in other cellular processes too (Morales et al., 2014). Several substrates of PARPs have been identified, and majority of them are nuclear proteins involved in processes like DNA repair and synthesis, chromatin remodeling, and nucleic acid metabolism (Amé et al., 2004). Because chromatin structure is extremely important for transcription, its regulation by PARylation is important. Matrix-binding proteins are important modulators of chromatin structure; hence, modulation of their function through PARPs is important. Interestingly, PARP1 itself is a major target of auto-PARylation, which in turn negatively modulates its DNA-binding ability (D'Amours et al., 1999). This serves as a means of negative feedback regulation to limit its activity only to the time frame required. Although PARPs have a similar mode of functions, their targets differ. PARylation plays an important role in DNA damage repair by helping in the recruitment of repair machinery to the sites of damage. Studies have shown SAFB1, a matrix attachment region-binding protein, is a component of the cellular response machinery to genotoxic stress. It is a target protein of PARP1. PARylation of SAFB1 transiently recruits it at the damage sites that helps in proper signaling and spreading of the phospho-y-H2AX marks in response to genotoxic stress (Altmeyer et al., 2013). Ku70 and DNA-PK are the core components of classical non-homologous end joining (NHEJ) pathway. Studies have shown that PARylation of Ku70 by PARP1 reduces the DNA-binding activity and promotes classical NHEJ pathway (Wang et al., 2006; Mansour et al., 2010; Cheng et al., 2011; Paddock et al., 2011). However, PARylation of DNA-PK catalytic domain enhances its kinase activity, thereby promoting its role in double-strand break repair (Ruscetti et al., 1998; Veuger et al., 2004). Ku70 competes with PARP1 for DNA binding, thus making classical NHEJ the preferred pathway to the backup NHEJ pathway. Interestingly, vertebrate Ku70 does not have PAR-binding domain. So PARP1 is initially recruited at the DNA damage sites. With increase in PARylation activity of PARP1, its auto-PARylation also increases, leading to lowering of DNA-binding affinity. Ku70 is predicted to interact with PARylated DNA-PK via pADPr motif (Pleschke et al., 2000; Gagne et al., 2008). PARylation of DNA-PK helps in its recruitment at the damage sites. Probably by this means, Ku70 is recruited at proper places. Therefore, under normal conditions, this mechanism ensures activation of classical NHEJ pathway over PARP1-dependent backup NHEJ pathway. Thus, this crosstalk between PARP1, PARylation, and Ku70 warrants further investigation. Another important class of S/MARBPs is the HMGI/Y/I-C proteins. These are of special interest, as they play important roles in several cellular processes like gene regulation and neoplastic transformation. Studies in HeLa cell line have shown that incubating isolated nuclei in Ca^2+^-containing buffer leads to PARylation of HMGI/Y/I-C proteins. This occurs in a similar fashion as it happens following DNA damage and apoptosis (Niedergang et al., 1979; Crabtree, 1989; Jones et al., 1989; Boulikas, 1990; Boulikas et al., 1990; Bachs et al., 1992; Bellomo et al., 1992; Realini and Althaus, 1992; Nicotera et al., 1994; Asher et al., 1995). Incubation of nuclei in Ca^2+^ buffers mimics conditions same as stimulation with signals like TNFa (Yang et al., 1993; Chaturvedi et al., 1994; Belka et al., 1995; Trump and Berezesky, 1995), which triggers cytosolic release of calcium from the endoplasmic reticulum. This cytosolic calcium enters nucleus-activating endonucleases (Green and Martin, 1995; Miyazaki, 1995). Apoptosis involves many cytosolic and nuclear processes, out of which disruption of Ca^2+^ homeostasis is one of those mechanisms. Therefore, calcium-induced PARylation of HMGI proteins might be related to the collapse of nuclear architecture during apoptosis (Earnshaw, 1995). Epigenetic information is coded in the form of DNA methylation patterns. MeCP2 is a DNA methylcytosine-binding protein, which modulates chromatin architecture. Experiments have demonstrated that MeCP2 binds chromatin with higher affinity in PARP–/– cells. That means PARylation of this protein reduces its affinity to chromatin (Becker et al., 2016). Thus, PARylation regulates MeCP2-mediated chromatin architecture, thereby modulating gene expression. In a recent study, it has been shown that CTCF and PARP colocalize to different regions across Epstein–Barr virus (EBV) genome. EBV is an oncogenic gamma herpesvirus that is responsible for 95% of the infections in humans worldwide. It infects memory B cells, circularizes to form episome, and establishes a chronic and latent infection in B cells. These latent infections are dangerous and lead to 1% of the human cancers. There are different types of latency seen in EBV (Lupey-Green et al., 2018). In type 0 latency, no viral gene is expressed in cells (Tempera et al., 2011). Type I latency occurs in Burkitt's lymphoma (BL), BL-derived cell lines, and memory B cells; and only EBNA-1 gene is expressed. In type III latency, all viral latency genes are expressed and is generally found associated with post-transplant and AIDS-related lymphomas. During latency period, EBV expresses specific sets of proteins depending on the type of latency it is in, and this is associated with the type of cancer. By doing ChIP-Seq studies using EBV-immortalized lymphoid cell lines (LCLs), the authors identified regions of the EBV genome where CTCF colocalizes with PARP1 and sites where either CTCF or PARP1 binds alone. PARP1 and CTCF together bind to the lytic promoter Zp, LMP1/2 promoters, and latency promoters Cp and Qp. Most interestingly, another ChIP for PAR using type I and type III latent cell lines clearly showed enrichment of PARylated CTCF at Cp latency promoter in type III latent cell line (Figure 6). Treatment with olaparib, a PARP inhibitor, led to loss of CTCF at Cp and changes in global binding of CTCF. PARylated CTCF is important for transcription from Cp latency promoter in type III latency-exhibiting cells. Olaparib led to a transition from type III to type I phenotype. Thus, these studies provide the rationale for using PARP inhibitors for use in treating type III latency-associated cancers (Lupey-Green et al., 2018). Another recent study highlights the role of CTCF180 form, which is the higher-molecular-weight PARylated form of CTCF. The hypo- or un-PARylated form of CTCF is designated as CTCF130. Though CTCF180 is present in ample amounts in primary tissues, its role is not fully elucidated. In this study, human breast 226LDM cells have been used, which display CTCF130 during proliferation and CTCF180 during cell cycle arrest. With the use of ChIP-Seq and RNA-Seq, it was observed that majority of the binding sites lost CTCF upon induction of cell cycle arrest, while some sites gained CTCF and some other common sites remained unchanged in terms of CTCF occupancy. The common sites and the ones that lost CTCF showed greater chromatin densities and altered expression of genes. Interestingly, gaining CTCF at new sites means that PARylation of CTCF leads to conformational changes that make it bind new sites. It is also worth questioning what other features demarcate two consensus sites such that one is recognized only when CTCF is PARylated while the other is recognized when not PARylated (Pavlaki et al., 2018). It is therefore evident that PARylation of S/MARBPs plays important roles in gene regulation, maintenance of chromatin architecture, and genomic stability.

## Conclusion and Future Perspectives

PTMs are one of the most important ways of modulating protein functions in living systems. It is evident that there exists crosstalk between PTMs of S/MARBPs, which regulate its activity in context-specific manner (summarized in Table 3). As discussed in this review, phosphorylation of SATB1 by PKC is enhanced in glioblastoma, which leads to increased association with HDAC1 (Han et al., 2013). Again, acetylation of SATB1 by PCAF at Lys136 leads to upregulation of its target genes (Purbey et al., 2009). Thus, modulation of the same S/MARBP by two different PTM leads to opposing functions. It would be interesting to see how these PTMs are altered in a disease scenario for each S/MARBP known to be involved. Not only the same S/MARBPs are modified using different PTMs, but also the same PTM controls two different S/MARBPs in the same situation. As mentioned earlier, Ku70 and PARP1 compete for DNA binding during DNA repair mechanism, depending on their PARylation status (Pleschke et al., 2000; Gagne et al., 2008). Initially, although PARP1 is recruited, auto-PARylation leads to its reduced DNA-binding ability. In parallel to this, Ku70 gets PARylated, which now occupies PARP1's position, thereby ensuring that classical NHEJ pathway is activated and does not back up NHEJ pathway. MUT p53 undergoes several PTMs that alter its oncogenic functions. For example, C-terminal acetylation of MUT p53 has been linked to autophagy under glucose restriction (Rodriguez et al., 2012). Now, this raises the question of how it is related to tumor growth under hypoxic conditions. As the tumor grows, the core becomes more and more hypoxic and necrotic. So it is expected that there the cells would be deprived of nutrients, and under such conditions, autophagy would be helpful. It would be interesting to see whether hypoxia alone triggers this C-terminal acetylation. Also, does this hold true for all the different MUT p53 types and across different origins of cancer? Another important task would be to check whether chemotherapy can also lead to C-terminal acetylation, providing either a pro-survival or pro-death cue via induction of autophagy. Like acetylation, there could be other PTMs of MUT p53 that might regulate this. p53 mutations are common to almost all cancers at some point of time, and so MUT p53 is now considered as the guardian of cancer genome. So efforts need to be made to identify the crosstalks between these PTMs to decipher how exactly these PTMs contribute and regulate the oncogenic functions of MUT p53. And targeting of MUT p53 would be a good option, as normal cells would not express that. Such studies would help in designing therapeutic interventions specific to cancer types. CTCF has also been shown to be de-SUMOylated under hypoxic conditions, leading to de-repression of P2 promoter of c-myc. In the context of cancer, this raises the question of whether any crosstalk exists between acetylated MUT p53 and de-SUMOylated CTCF under hypoxic conditions. Hypoxia following a stroke is common and leads to brain damage (Ferdinand and Roffe, 2016). The role of such post-translationally modified S/MARBPs needs to be studied under such condition to help in damage prevention. As mentioned before, CTCF is also PARylated, and one of the interesting functions of PARylated CTCF is to help in the maintenance of EBV latency in B cells. Now the questions come of which other viral latencies might it regulate and what other S/MARBPs are involved in this. HIV is one of the deadliest viruses affecting mankind that leads to AIDS. HIV-infected individuals require life-long antiretroviral therapy to survive. Within few days following infection, latency is established in CD4^+^ T cells. There exists a heterogeneous population of latently infected CD4+ T cells. Usually, these latent cells are activated by T-cell receptor stimulation by anti-CD3/anti-CD28 to clear the load of infected cells, but only a small population of latently infected CD4+ T cells responds (Rezaei et al., 2018). This highlights the need for identifying other mechanisms that might regulate this process. PARylated CTCF has been already found to regulate latency in EBV, so it may be investigated whether it also has any role in the maintenance of HIV latency. It may be so that some other post-translationally modified form of CTCF may be involved. Interestingly, >98% of HIV sequences contain an S/MAR element. And studies from our lab have shown that SMAR1 binds to the HIV long terminal repeat (LTR) S/MAR and reinforces transcriptional repression (Sreenath et al., 2010; Trivedi et al., 2020). It would be interesting to see if this involves any PTM and if targeting of that PTM may be used to break the latency. There could be many more S/MARBPs involved that need to be identified to develop newer strategies to tackle these constantly evolving viruses. These examples nicely demonstrate how PTMs of S/MARBPs orchestrate their cellular functions. Tissue-specific PTMs would also impart organ-specific functions of same S/MARBP. Still, many more aspects need to be uncovered regarding the regulation of S/MARBPs. Only a limited number of examples of PTMs are available in literature; many more are yet to be reported like prenylation and farnesylation. Recently, serotonylation of histone proteins has been identified (Farrelly et al., 2019). It would be interesting to see if such modification occurs on S/MARBPs as well. A relatively recent study has used ubiquitin-specific antibody designated as UbiSite for mapping the lysine and N-terminal ubiquitinations. Using high-accuracy MS, they identified 63,000 unique ubiquitination sites on 9,200 proteins including S/MARBPs in Hep2 and Jurkat cell lines. Interestingly, they found an inverse correlation between ubiquitination and acetylation. However, there was no correlation between protein levels and changes in the ubiquitination sites upon inhibiting proteasomal degradation (Akimov et al., 2018). These results indicate not only crosstalk between different PTMs but also non-canonical functions of ubiquitination. More such large-scale studies are necessary to understand the functions and tissue-specific roles of PTM. Since the major function of S/MARBPs is to alter chromatin structure, it is worth questioning if they might regulate secondary DNA structures like G-quadruplexes, which play important roles in the regulation of transcription. One good example of an S/MARBP binding to G-quadruplex structures is nucleolin (González et al., 2009). It needs to be studied if any PTM of nucleolin might affect that. Several S/MARBPs act as tumor suppressors; one good example is SMAR1, which is found to be downregulated in majority of cancers. Inhibitors may be designed in a way such that the detrimental PTMs can be prevented. S/MARBPs can also regulate each other to promote certain outcomes. A very recent study reports transcriptional regulation of hnRNP K by MUT p53 R175H, which is a hotspot mutant form of p53. Pancreatic ductal adenocarcinoma (PDAC) is driven by coexisting mutations in both KRAS and p53. MUT p53 R175H transcriptionally upregulates hnRNP K, which in turn promotes the expression of alternatively spliced isoforms of GTPase-activating proteins (GAPs), negative regulators of RAS family members that lose their membrane association. This causes heightened KRAS signaling (Escobar-Hoyos et al., 2020).

**Table 3:** Post translational modification of known MARBPs in eukaryotes.

**Target MARBP**	**Function**	**References**
**Phosphorylation**		
SATB1	PKC Phosphorylates SATB1 leading to increased association with HDAC1 resulting in altered gene expression	Ning et al., 2020
Drosophila and mammalian cut homeodomain proteins	PKC phosphorylates cut homeodomain repeats 1,2, 3 at Thr415, Thr804, Ser987, respectively, reducing DNA binding ability	Han et al., 2013
Murine cut homeodomain proteins	CKII phosphorylates cut homeodomain repeats 1, 2, 3 at Ser400, Ser789, Ser972, respectively, decreasing DNA binding ability	Coqueret et al., 1996
p110 Cux/CDP	Cyclin A/cdk1 phosphorylates p110 Cux/CDP at Ser1237 and Ser1270 in G2 phase of cell cycle resulting in reduced DNA binding activity	Coqueret et al., 1998
Lamin B	PKCα phosphorylates Lamin B in HL60 cells resulting in proteolysis of Lamin B and subsequent DNA fragmentation	Zaremba-Czogalla et al., 2012
GRASS404 sequence of mammalian Lamin C	PKC phosphorylates mammalian Lamin C and is important for its nuclear import which then plays a role maintenance of nuclear architecture	Eggert et al., 1993; Leukel and Jost, 1995; Shimizu et al., 1998
Chicken Lamin B2	Ser410/Ser411 of Chicken Lamin B2 is phosphorylated by PKC that regulates its import into nucleus that then plays a role in maintenance of nuclear architecture	Haas and Jost, 1993
MeCP2	Mutational analysis in Rett's syndrome indicated a role for MeCP2 phosphorylation at Ser80 in modulating its association with chromatin in resting neurons. However, calcium influx causes dephosphorylation causing dissociation.	Hennekes et al., 1993
MeCP2	Membrane depolarization caused Ser421 phosphorylation of MeCP2 leading to bdnf promoter activation in neuronal cells by reducing MeCP2 DNA binding activity	Tao et al., 2009
AcMFP1	CKII phosphorylates AcMFP1 in a cell cycle dependent manner resulting in reduced nuclear matrix binding	Zhou et al., 2006
Chloroplast localized MFP1 in tobacco plants	CKII phosphorylates chloroplast MFP1 to decrease association with the chloroplast nucleoid	Cohen et al., 2011
HMGA1a	Cdc2 phosphorylates Thr52, Thr77, Ser35 of HMGA1a *in vitro* and in metaphase arrested cells leading to reduced DNA binding ability	Nissen et al., 1991; Meier et al., 1996; Samaniego et al., 2006
HMGA1a	HIPK2 phosphorylates Thr52, Thr77, Ser35 of HMGA1a leading to reduced DNA binding ability at the human germline ε promoter.	Nissen et al., 1991
HMGA1a	PKC phosphorylates HMGA1a at Ser43 and Ser63 and attenuates binding with PKC⋎ and neurogranin/RC3 promoter	Reeves et al., 1991
HMGA1a	Hyperphosphorylation of HMGA1a has been seen in early apoptotic leukemia cells U937, K562, HL60, NB4 leading to displacement from chromatin that helps playing a role in formation of early apoptotic bodies containing condensed chromatin. The ATM kinase pathway plays a role.	Zhang and Wang, 2007
HMGA1b	ATM kinase phosphorylates at Ser87 within the SQ motif in response to DNA damage and leads to chromosomal reorganization with changes in gene expression.	Xiao et al., 2000
Nucleolin	Protein Kinase NII phosphorylates nucleolin that leads to increased ribosomal biogenesis in fetal bovine arterial endothelial cells.	Diana et al., 2001
SMAR1	SMAR1 is phosphorylated by ATM kinase at Ser370 which helps in recruitment of deacetylated Ku70 at DNA double stranded breaks ensuring timely repair	Pentimalli et al., 2008
MUT P53	The N terminus of MUT P53 is phosphorylated by JNK and C-terminus is phosphorylated by PLK2 both leading to enhanced oncogenic activities. Ser392 was phosphorylated in MUT P53 leading to tetramer stabilization and increased DNA binding activity leading to oncogenic properties	Ullrich et al., 1993; Minamoto et al., 2001; Matsumoto et al., 2004a
CTCF	CTCF is phosphorylated by PLK1 at Ser224 promoting binding at a subset of CTCF binding sites and affects changes in gene expression including upregulation of p21 and p53 during G2/M transition in mouse embryonic stem cell colonies.	Matsumoto et al., 2004a
**Acetylation**		
SATB1	Acetylation of SATB1 by PCAF at Lys136 in PDZ domain leads to disruption of interaction with CtBP1 leading to upregulation of target genes.	Yap et al., 2004
HMGIY	Lys71 is acetylated by PCAF/GCN5 that activates transcription of IFNβ by stabilizing the enhanceosome complex while acetylation at Lys65 by CBP destabilizes this complex leading to downregulation	Bode and Dong, 2004
Ku70	An increased Ku70 acetylation in the aggressive form of neuroblastoma N type compared to less aggressive type S reduces the DNA binding ability of Ku70	Luo et al., 2020
MUT P53	Arg273His and Arg248Trp mutants of P53 are hyperacetylated Lys320, Lys373, Lys382 that helps in nuclear accumulation	Chaudhary et al., 2014
MUT P53	CBP/P300 mediated acetylation of Lys373 under Id4 overexpression in DU145 cell line promoting DNA binding activity	Li et al., 2000
**Sumoylation**		
SATB2	This is sumoylated by PIAS1 at Lys233 and Lys350 downregulating DNA binding activity at immunoglobulin μ gene in pre-B cells.	Tang et al., 2008
SAFB1	SAFB1 is SUMO1 labeled by PIAS1 and is important for RNA Pol II recruitment at ribosomal protein and translator genes. Sumoylated SATB1 acts as transcriptional activator.	Knowell et al., 2013
SAFB1	Interestingly SAFB1 is modified by SUMO1 and SUMO2/3 at Lys231 and Lys294, respectively. This modification makes it act like a co-repressor activity.	Rodriguez et al., 2012
Ku70	Ku70 is Sumoylated at five residues in its C-terminal tail Lys588, Lys591, Lys592, Lys595, and Lys596 thereby promoting DNA binding activity during DNA repair and telomere maintenance	Perez et al., 2010
MeCP2	It is sumoylated at Lys223 that is required for transcriptional repression activities. It helps in recruitment of HDAC1/2 complex. In rats abrogating this site leads to poor hippocampal synapse development in rats thus highlighting its importance in central nervous system development.	Dobreva et al., 2003
CTCF	CTCF is sumoylated by SUMO1, 2, 3 both N-terminal and C-terminal domain by Pc2 E3 ligase. This is important for repression of c-Myc P2 promoter.	Liu et al., 2015
CTCF	CTCF is desumoylated at Lys74 and 689 by SENP1 under hypoxic conditions in human corneal cells	Garee et al., 2011
**Methylation**		
HMGA1a	Arg25 of the first AT hook in HMGA1a is found to be methylated in human leukemia cells, rat thyroid tumors cells, human prostate tumor cells during apoptosis. *In vivo* PRMT1 methylates HMGA1a at this site.	Cheng et al., 2014; Hang et al., 2014
HMGB1	In neutrophils HMGB1 is methylated at Lys42 leading to weakening of DNA binding ability causing export to cytoplasm.	MacPherson et al., 2009
WTP53	WTP53 is methylated at Lys370 by SMYD2 causes repression of activity while methylation at Lys372 by SET7/9 causes stabilization of chromatin associated SMAR1	Sgarra et al., 2003; Wang et al., 2012
**PARylation**		
PARP1	PARP1 auto-PARylates itself that leads to reduced DNA binding ability	Scott et al., 2012
SAFB1	SAFB1 PARylation helps in recruitment of p-⋎-H2AX at damage sites in response to DNA damage responses	West and Gozani, 2011
Ku70	Ku70 is PARylated that reduces DNA binding activity and promotes classical NHEJ pathway.	D'Amours et al., 1999; Amé et al., 2004; Altmeyer et al., 2013; Morales et al., 2014
DNA-PK catalytic domain	PARylation of DNA-PK catalytic subunit increases its kinase activity thereby enhancing its role in double stranded DNA break repair.	Mansour et al., 2010; Cheng et al., 2011
HMGI/Y/I-C	Incubation of isolated HeLa cell nuclei in calcium containing buffers lead to PARylation of HMGI/Y/I-C proteins leading to probable collapse in nuclear architecture	Niedergang et al., 1979; Jones et al., 1989; Boulikas, 1990; Boulikas et al., 1990; Realini and Althaus, 1992; Asher et al., 1995; Belka et al., 1995; Ruscetti et al., 1998; Pleschke et al., 2000; Veuger et al., 2004; Gagne et al., 2008
MeCP2	MeCP2 has lower chromatin binding affinity in PARP-cells therefore substantiating the fact that PARylation of MeCP2 leads to reduced DNA binding affinity	Trump and Berezesky, 1995
CTCF	PARylated CTCF is enriched at the Cp promoter in type III latency exhibiting EBV immortalized cell line testifying its importance in transcription	Miyazaki, 1995
CTCF	Studies using human breast 226LDM cells it has been found that several new sites are recognized by CTCF on PARylation	Earnshaw, 1995

**Figure 7:**
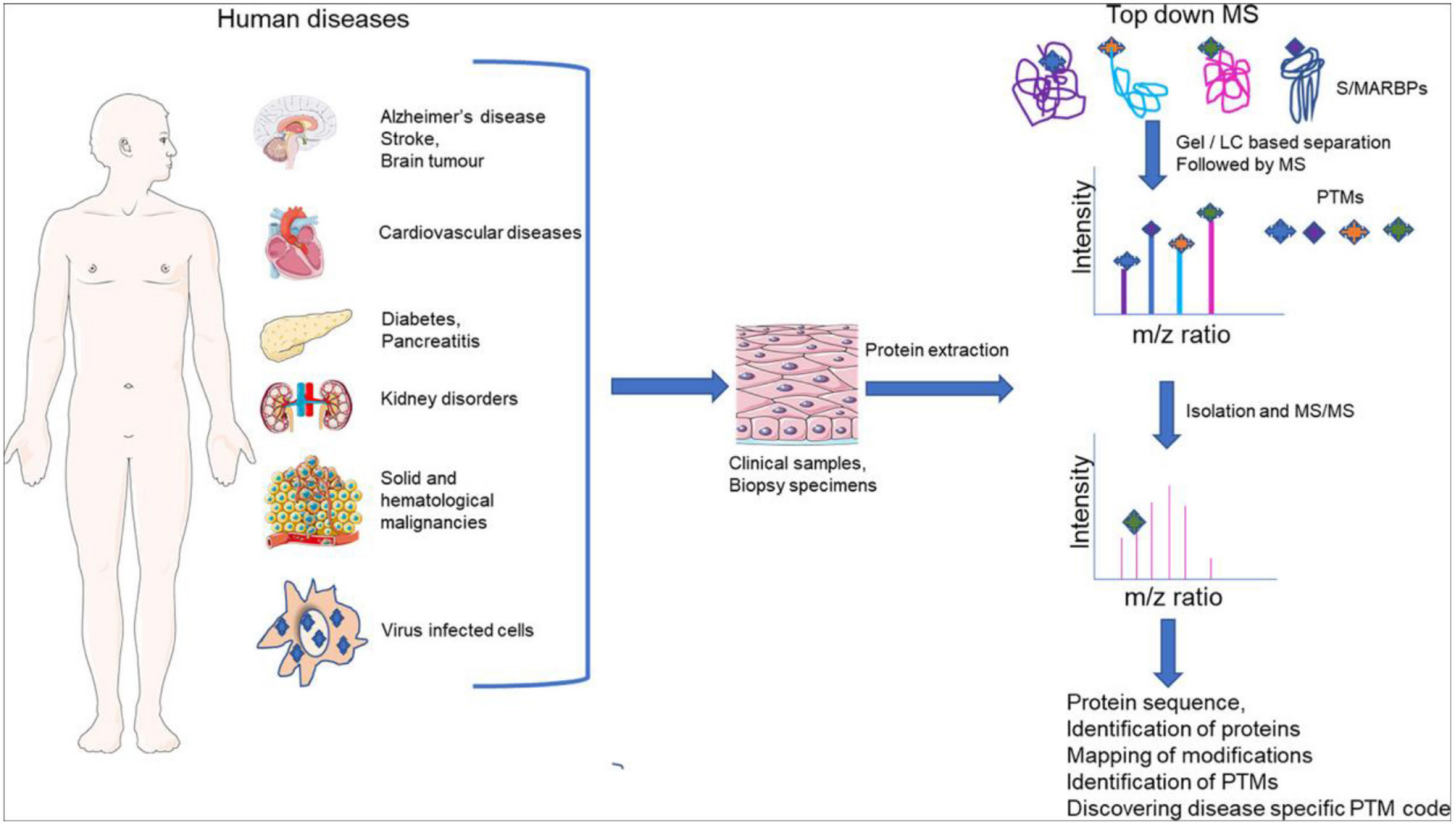
Identification of link between S/MARBP PTM code and disease predisposition.

So it is important to understand how PTMs might regulate important S/MARBPs under diverse cellular situations. It would be exciting if we can identify disease-specific patterns of S/MARBP PTMs like fingerprints. PTMs that are more prominent in a disease can be used as biomarkers, if specific. For example, in a disease like cancer, if gradewise predominance of a single or an array of S/MARBP PTMs exists, then it would be helpful in assessing biopsy specimens. In present times, multiple diseases like type 2 diabetes, Alzheimer's disease, obesity, cancer, polycystic ovarian syndrome, and cardiovascular disorders are on the rise due to a combination of contributing factors like genetics, epigenetics, food habits, environmental factors, and lifestyle. The roles played by S/MARBPs and their PTMs in development and progression of these diseases remain a black box and warrant further investigation. Table 4 summarizes the list of some disorders associated with mutations in the S/MARBPs using the Human Gene Mutation Database (HGMD, Stenson et al., 2017) and Catalog of Somatic Mutations in Cancer (COSMIC, Tate et al., 2019). Identification of any link between S/MARBP PTM predominance and disease predisposition would be useful in earlier detection of diseases. Also, it may be used to map a response to treatment strategies. In recent years, top-down MS has been used to evaluate protein biomarkers, metabolites, and PTMs in clinical samples like body fluids and tissues (Calligaris et al., 2011). Matrix-assisted laser desorption/Ionization (MALDI) top-down MS is also used for diagnostic imaging of breast cancer tissue resections for proper evaluation of HER2 receptor classification status (Rauser et al., 2010). These techniques may be used to deduce S/MARBP PTM code specific to a disease (Figure 7). It would not only allow for identification of PTMs but also highlight location-specific enrichment of PTMs and crosstalk with other proteins or PTMs. Regardless of the disease, every patient is unique, and thus, the concept of personalized therapy has emerged. It would be interesting to see how such S/MARBP PTMs would be useful in the formulation of personalized therapeutic strategies.

**Table 4:** Disease causing reported mutations in S/MARBPs.

**Protein**	**Mutation**	**Phenotype**	**Source**
CUX1	Gln21Term	Developmental delay	HGMD
	Gln873Term	Developmental delay	HGMD
	Ala3Asp	Stomach adenoarcinoma	COSMIC
	Gln10Glu	Endrometroid carcinoma	COSMIC
	Leu17Phe	Colon adenocarcinoma	COSMIC
	Asp25Val	Ovarian serous carcinoma	COSMIC
	Ala28Thr	Prostate carcinoma	COSMIC
	Thr29Ala	Lung adenocarcinoma	COSMIC
	Arg44Trp	Liver carcinoma	COSMIC
	Pro57Ser	Melanoma	COSMIC
SMAR1	Lys87Gln	Keratoconus	HGMD
	Ile92Leu	Keratoconus	HGMD
	Ala153Thr	Congenital heart disease	HGMD
	Asp6Asn	Colon adenocarcinoma	COSMIC
	Arg246His	Tongue squamous cell carcinoma	COSMIC
	Leu18Met	Endometroid carcinoma	COSMIC
	Glu27Gly	Lung carcinoma	COSMIC
	Val31Leu	Ovarian serous carcinoma	COSMIC
	Gln49Lys	Breast carcinoma	COSMIC
	Arg126Trp	Cervical squamous cell carcinoma	COSMIC
CTCF	His373Leu, Lys206Glu	Endometriosis	HGMD
	Arg278Leu, Arg283His, Arg339Gln, Arg342Cys, Arg342His, Arg368His, Arg377His, Arg448Gln, Arg654Term, Asp390Asn, Asp529Asn, Cys327Ser, Cys409Tyr, Glu336Gln, His373Asp, His373Pro, Ser360Arg	Neurodevelopmental disorder	HGMD
	His294Pro	Intellectual disability/developmental delay	HGMD
	Tyr343Cys	Abnormality of the nervous system	HGMD
	Arg368Cys, Pro378Leu	Developmental disorder	HGMD
	Arg368Leu	Mental retardation, autosomal dominant 21	HGMD
	Tyr343Cys	Abnormality of the nervous system	HGMD
	Arg415Gln	Tourette syndrome	HGMD
	Glu2Val	Breast carcinoma	COSMIC
	Val6Ile	Burkitt lymphoma	COSMIC
	Ser13Phe	Skin basal cell carcinoma	COSMIC
	Glu14Lys	Lung adenocarcinoma	COSMIC
	Arg28Cys	Astrocytoma grade IV	COSMIC
	Arg29Gln	Clear renal cell carcinoma	COSMIC
	Asp46Asn	Ovarian serous carcinoma	COSMIC
	Met63Ile	Hepatocellular carcinoma	COSMIC
Ku70	Met348Th, Gly589Arg	Amyotrophic lateral sclerosis	HGMD
	Glu11Gln	Breast carcinoma	COSMIC
	Gln65Lys	Renal clear cell carcinoma	COSMIC
	Phe87Leu	Endometroid carcinoma	COSMIC
	Thr90Ile	Melanoma	COSMIC
	Ala113Val	Oligodendroglioma grade III	COSMIC
	Gly197Arg	Ovarian serous carcinoma	COSMIC
	Asp241Tyr	Prostate carcinoma	COSMIC
	Arg258Trp	Gall bladder carcinoma	COSMIC
	Pro285Thr	Plasma cell myeloma	COSMIC
MeCP2	Met1Leu, Ala3Pro, Lys22Term, Gln47Term, Ser49Term, His51Gln, Ser65Term, Ala59Pro, Asp97Tyr, Leu100Arg, Pro101His, Arg106Gly, Leu108His, Tyr120Asp, Asn126Ser, Arg133Leu, Phe157Leu, Pro322Thr	Rett syndrome	HGMD
	His52Gln	Hypotonia, neonatal	HGMD
	Gly69Arg, Ala73Pro	Anorectal malformation	HGMD
	Leu108Pro	Seizures, microcephaly, hypotonia and multiple congenital anomalies	HGMD
	Gly118Glu	Cognitive impairment	HGMD
	Val122Ala	Learning disability with progressive ataxia, spasticity and dystonia	HGMD
	Glu137Gly, Asp147Glu, Arg167Trp, Arg453Gln, Pro405Leu, Pro403Arg, Pro399Leu, Arg309Trp	Mental retardation, X-linked	HGMD
	Tyr141Ser	Epilepsy, early-onset	HGMD
	Tyr141Term	Angelman syndrome	HGMD
	Pro152Ala, Pro152Leu	Autism spectrum disorder	HGMD
	Phe157Ile	Neonatal encephalopathy, severe	HGMD
	Thr160Ser, Pro376Arg,	Autism	HGMD
	Ser164Ile	Asperger syndrome & schizophrenia, early-onset	HGMD
	Arg168Gln	Parkinsonism, intellectual disability & catatonia	HGMD
	Pro176His	Progressive encephalopathy	HGMD
	Ala181Val	Infantile autism	HGMD
	Arg190Cys, Thr196Ser	Schizophrenia	HGMD
	Lys304Thr	Epilepsy and/or neurodevelopmental disorders	HGMD
	Lys305Asn	Focal epilepsy, drug-resistant	HGMD
	Pro322Ser	Mental retardation and progressive spasticity	HGMD
	Arg478Gln	Stomach carcinoma	COSMIC
	Met466Leu	Hepatocellular carcinoma	COSMIC
	Arg453Leu	Lung adenocarcinoma	COSMIC
	Asp427Asn	Oesophagial squamous cell carcinoma	COSMIC
	Met418Ile	Endometroid carcinoma	COSMIC
	Ser360Leu	Cervical squamous cell carcinoma	COSMIC
Nucleolin	Gln569Term	Developmental disorder	HGMD
	Gly677Asp	Stomach adenoarcinoma	COSMIC
	Arg673Gln	Oesophagial carcinoma	COSMIC
	Gly649Val	Hepatocellular carcinoma	COSMIC
	Lys648Thr	Colon adenocarcinoma	COSMIC
	Ala645Val	Head and neck squamous cell carcinoma	COSMIC
	Met630Ile	Pancreatic ductal carcinoma	COSMIC
	Arg604Gln	Cervical squamous cell carcinoma	COSMIC
	Glu585Lys	Bladder carcinoma	COSMIC
	Glu547Gly	Nasopharyngeal carcinoma	COSMIC
PARP1	Phe54Leu	Diabetic polyneuropathy, reduced risk, association with	HGMD
	Gln150His, Ser383Tyr, Arg452Arg	Breast cancer	HGMD
	Ala284Ala	Colorectal adenoma, association with	HGMD
	Leu293Phe	Intellectual disability	HGMD
	Pro605Leu, Val886Met	Melanoma	HGMD
	Val762Ala	Prostate cancer, susceptibility, association with	HGMD
	Lys933Asn, Lys940Arg, Lys945Asn	Colorectal cancer, increased risk	HGMD
	Ser1012Phe	Melanoma	COSMIC
	Ala995Asp	ER +ve breast carcinoma	COSMIC
	Asp965Asn	Lung adenoarcinoma	COSMIC
	Val948Phe	Thyroid carcinoma	COSMIC
	Asp906Ser	Hepatocellular carcinoma	COSMIC
	Ser904Thr	Colon adenocarcinoma	COSMIC
SATB1	Asp763Gly	Hepatocellular carcinoma	COSMIC
	Asp765Tyr	Endometroid carcinoma	COSMIC
	Leu745Val	Stomach carcinoma	COSMIC
	Glu729Lys	Bladder carcinoma	COSMIC
	Glu718Gly	Lung carcinoma	COSMIC
	Phe662Ser	Prostate carcinoma	COSMIC
	Gln596Arg	Thyroid	COSMIC
	Glu530Lys	Acute lymphoblastic T cell leukemia	COSMIC
	Ile464Met	Cervical squamous cell carcinoma	COSMIC
	Ala455Val	Pancreatic ductal carcinoma	COSMIC
SATB2	Gly42Term, Gly116Arg, Gln419Term, Arg429Gln, Pro655Leu	Developmental delay with severe speech impediment, facial dysmorphism & dental anomalies	HGMD
	Val62Asp, Leu86Arg, Leu96Arg, Gln250Term, Arg283Term, Gln290Term, Gln330Term, Gln333Term, Val368Phe, Gln379Pro, Gln379Term, Thr390Ile, Gln391Term, Gly392Arg, Gly392Glu, Arg399Leu, Arg399Pro, Gln514Arg	SATB2-associated syndrome	HGMD
	Arg429Term, Arg399His, Leu394Ser, Arg399His, Leu394Ser, Gly515Ser, Glu566Lys	Intellectual disability, syndromic	HGMD
	Ile621Phe, Pro655Ser	Autism spectrum disorder	HGMD
	Lys499Term	Moderate intellectual disability, speech delay, hypotonia, crowded teeth and brain abnormalities	HGMD
	Arg532Cys	Anencephaly	HGMD
	Arg389His	Glass syndrome	HGMD
	Met293Ile, Gln573His	Marfan syndrome	HGMD
	Ala607Glu	Breast ductal carcinoma	COSMIC
	Glu600Ala	Endometroid carcinosaroma	COSMIC
	Glu590Lys	Melanoma	COSMIC
	Glu586Lys	Lung squamous cell carcinoma	COSMIC

Although technological advancements have enabled the detection of such PTMs, there are several challenges that are yet to be addressed. To understand the genome-wide function of a PTM-modified S/MARBP, it is a prerequisite to have specific antibodies against a particular PTM-modified S/MARBP. But developing a highly potent and specific antibody against a PTM is difficult for various reasons. These chemical modifications on protein are most of the time very minute, and subtle differences exist across the different PTMs. Sometimes, antibodies are required against a PTM only or both the PTM and the surrounding sequences. All these make generation of specific antibodies against PTMs a formidable challenge (Hattori and Koide, 2018). Many a time, the antibodies fail in specificity testing. For example, ~over 25% of more than 200 commercially available antibodies against histone PTMs failed in specificity testing (Egelhofer et al., 2011). Apart from these issues, one major hurdle is that majority of the antibodies available for research purpose are polyclonal in nature and are therefore not reproducible (Hattori and Koide, 2018). Accessibility of the PTM site within a protein is also an important factor that affects antibody generation. At any given time, only a certain fraction of any protein is post-translationally modified, thus requiring enrichment strategies. Several methods have been developed such as immunoaffinity chromatography, immobilized metal ion affinity chromatography (IMAC), and metal oxide affinity chromatography (MOAC); however, these techniques also have specificity issues. However, numerous strategies are being developed to overcome the issue (Virág et al., 2020). PTMs are, most of the time, highly labile in nature, and this makes detection even more difficult (Smith and Rogowska-Wrzesinska, 2020). Since only a small fraction of a particular S/MARBP would be modified in response to a stimulus, a ChIP-Seq analysis against a post-translationally modified PTM would lead to low read counts, depending on binding affinities and genome-wide binding site distribution, leading to loss of information. These represent some of the present challenges in this field of chromatin biology.

However, with continued technological improvements, it is expected that more clinically valuable information would pour in. Although still in its infancy, gaining an in-depth knowledge will help us to design better therapeutic strategies and have a strong impact on clinical research and health.

## Author Contributions

TR wrote the original draft. TR and SC contributed to the conceptualization of the study, provided supervision, reviewed, and edited the paper. SC acquired the funding. All authors contributed to the article and approved the submitted version.

## Conflict of Interest

The authors declare that the research was conducted in the absence of any commercial or financial relationships that could be construed as a potential conflict of interest.
